# Sensing Biomolecules Associated with Cells’ Radiosusceptibility by Advanced Micro- and Nanospectroscopy Techniques

**DOI:** 10.1021/acssensors.4c01455

**Published:** 2024-09-18

**Authors:** Karolina Chrabąszcz, Katarzyna Pogoda, Klaudia Cieżak, Agnieszka Panek, Wojciech M. Kwiatek

**Affiliations:** Institute of Nuclear Physics Polish Academy of Sciences, Radzikowskiego 152, 31-342 Krakow, Poland

**Keywords:** spectroscopic detection, radiosusceptibility, microspectroscopy, nanospectroscopy, atomic force microscopy

## Abstract

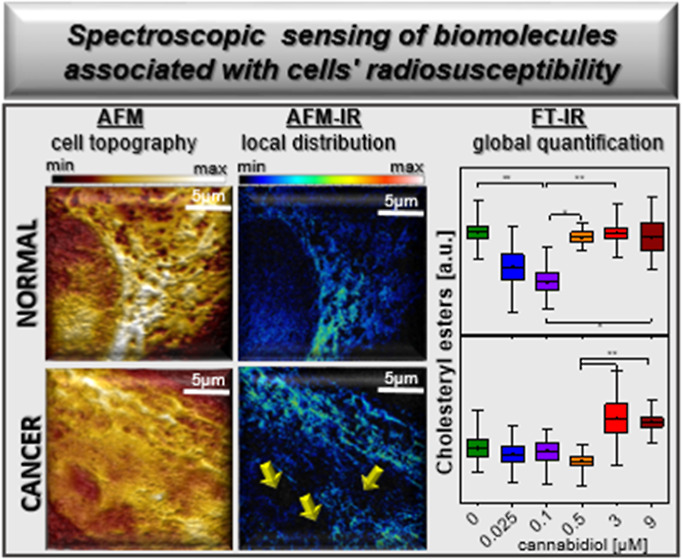

Radiotherapy is one of the most common approaches for cancer treatment, especially in the case of peripheral nervous system tumors. As it requires exposure to high doses of ionizing radiation, it is important to look for substances that support efficient reduction of the tumor volume with simultaneous prevention of the surrounding noncancerous cells. Cannabidiol (CBD), which exhibits both anticancer and neuroprotective properties, was applied as a potential modulator of radiological response; however, its influence on cells undergoing irradiation remains elusive. Here, we have applied high-resolution optical spectroscopy techniques to capture biomolecules associated with CBD shielding of normal and damaging cancerous cells upon X-ray exposure. Conventional Raman (RS) and Fourier transformed infrared (FT-IR) spectroscopies provided semiquantitative information mainly about changes in the concentration of total lipids, DNA, cholesteryl esters, and phospholipids in cells. A through assessment of the single cells by atomic force microscopy coupled with infrared spectroscopy (AFM-IR) allowed us to determine not only the alterations in DNA content but also in its conformation due to cell treatment. Pronounced nanoscale changes in cholesteryl ester metabolites, associated with CBD treatment and radiation, were also observed. AFM-IR chemoselective maps of the single cells indicate the modified distribution of cholesteryl esters with 40 nm spatial resolution. Based on the obtained results, we propose a label-free and fast analytical method engaging optical spectroscopy to assess the mechanism of normal and cancerous cell susceptibility to ionizing radiation when pretreated with CBD.

## Introduction

Cancer remains in the top three of the deadliest diseases worldwide; moreover, it is estimated that in 2060, it will be the leading cause of death.^1^ Although innovative anticancer therapies are under investigation, radiotherapy is still one of the most used treatment for various cancer types, including peripheral nervous system (PNS) tumors.^2−4^ Since radiation therapy uses high doses of ionizing radiation, it is important to look for a treatment that allows for the efficient reduction of the tumor volume while maintaining the normal functions of the surrounding cells and decreases the overall side effects. Cannabidiol (CBD) is a promising compound that could exhibit such two-pronged approach by increasing the toxicity of ionizing radiation in tumors with simultaneous protection of surrounding normal cells.^5,6^ However, up until now the alterations in cell biochemistry underlying such modulatory mechanisms remain elusive.

Commonly, to address an issue related to unknown biochemical responses in cells related to, for example, effectiveness of potential drugs, the introduction of multiple quantitative methods is necessary. Typically, to determine changes in protein and lipid fractions, Western blot and liquid chromatography are implemented, as well as RT-PCR for DNA sequencing, or fluorescent staining to visualize cell morphology and its subcellular components.^7−9^ Yet, these methods require the use of expensive chemical reagents during complicated multistage processes which is usually laborious and leads to changes in the studied sample.

Reaching the need for obtaining rapid and label-free outcomes, the Raman (RS) and infrared (IR) spectroscopy techniques are often engaged as an objective and fast analytical methods.^10,11^ When coupled with microscopy, these methods do not require complex sample preparation and can obtain micro- or even nanoscale resolution. Acquired chemical maps allow for monitoring not only the total chemical composition of a biological sample but also the distribution of molecules even at the subcellular level. The combination of chemical information with bright field or topography images of the cells or tissues can lead to comprehensive analysis of the disease state or therapeutic effects due to treatment.^12−16^ In comparison to conventional analytical methods for biomolecular sample characterization, which may take a few days, the collection of chemical maps with spectral database usually takes up to 30 min per sample.

Despite many advantages being offered by Raman and FT-IR, their spatial resolution is diffraction-limited. Depending on the microscope objective (magnification and numeric aperture) and the wavelength of incident light, in general, it is possible to reach a spatial resolution of ∼1 μm. To overcome this limitation, the combination of atomic force microscopy (AFM) with the IR technique (AFM-IR) was introduced. This method enables for the investigation of biochemical changes at the nanoscale and with the accurate representation of the topography of the studied sample.^17,18^ Unlike the conventional FT-IR, where the spectra are directly generated based on light absorption, AFM-IR takes advantage of the samples photothermal expansion due to light absorption. It is induced by pulsed IR laser directed on the sample which absorbs the laser light what causes thermal expansion detectable by AFM tip.^19^ Therefore, the spatial resolution in lateral direction of AFM-IR system is limited by the diameter of AFM tip used for the study, which in our case is ca. ∼30 nm in the contact mode.^20^ The application of the tapping mode, which induces the rapid oscillation of AFM cantilever, allows to reach ∼15 nm lateral resolution.^21^ In the case of the sampling depth, the tip penetration depends both on sample stiffness and measurement mode (contact, tapping, or surface sensitive). For rigid materials, the probing depth will be lower than that for softer ones indented with the same nominal force. Since AFM-IR technique uses photothermal expansion of the whole sample volume, for cells measured in the contact mode the sampling depth amounts approximately 300 nm and relates to the height of the dried cell. For the tapping mode, where the AFM tip oscillates over the sample, it is more superficial and amounts ∼50 nm.^22^ However, the introduction of new AFM-IR measurement mode called surface sensitivity mode, based on force modulation microscopy, has the potential to achieve the probing depth of ∼25 nm.^23^

Since collected databases contain thousands of spectra per single cell, the analysis based on clustering is useful for constraining their number. Cluster analysis (CA) allows for spectral grouping established on the spectral profiles’ similarity. In case of hyperspectral images obtained for cells, such CA permits to differentiate map area occupied by whole cell as well as distinguish subcellular component as nuclei, cytoplasm, endoplasmic reticulum and lipid droplets accompanied by spectra characteristic for the particular group.^24,25^ Therefore, it is possible to correlate the unique spectral pattern with an accurate localization within the cell area. Moreover, obtained Raman, FT-IR, and AFM-IR spectra reflect the biochemical composition of the investigated sample where the band intensity is directly proportional to the amount of biomolecules.^26,27^ By calculation of band integral intensities, the detailed characterization of changes in the biochemical composition can be performed, as semiquantitative analysis.

Herein, for the first time, we propose the use of hyperspectral imaging for qualitative and quantitative assessment of biomolecules that undergo modification under the influence of CBD during the radiotherapy of PNS tumors.^28^

## Materials and Methods

### Cannabidiol

CBD solution (1.0 mg/mL) in methanol was purchased from Merc. The solution was initially dissolved in methanol to obtain the 1000 μM concentration and stored at −20 °C. It was further diluted in a cultured medium to the desired concentrations for cell studies. The dilutions were made considering the methanol concentration below 0.001%, to exclude methanol toxicity on cells. Spectral characterization of CBD can be found in the Supporting Information, Figure S1.

### Cell Culture

Studies were conducted on two cell lines purchased from ATCC: Human Schwann cells isolated from the peripheral nerve trunk (normal, hTERT NF1 ipnNF95.11c) and human MPNST derived from lung metastasis (cancer, sNF02.2). More details of the cell’s cultivation can be found in the Supporting Information.

### MTS Assay

To investigate CBD influence on the cells viability and metabolic activity, the cells were tested after 24 h of incubation with CBD using CellTiter 96 AQueous One Solution Cell Proliferation Assay (Promega) with tetrazolium compound. More details can be found in the Supporting Information.

### Irradiation Procedure

For irradiation studies, cells were seeded on calcium fluoride windows (CaF_2_) (Crystran Ltd., UK) inside 12-well plates and kept in an incubator at 5% CO_2_ and 37 °C for 24 h to promote adhesion and growth. The cell confluence after that time was ca. 70%. For each cell line, two sets of samples were prepared, as presented in Figure S2. The irradiation with a single fraction of X-rays at a dose rate of 2.1 Gy min^–1^ was performed using an MG325 (250 kV, 10 mA) X-ray tube (YXLON, Hamburg, Germany). More details can be found in the Supporting Information.

### Comet Assay

Comet assay was applied to analyze the genotoxic effect of CBD and CBD in combination with X-ray radiation. DNA damage levels were carried out directly after 24 h incubation with CBD and after 24 h incubation with CBD and irradiation using the alkaline version of the comet assay. More details of the comet assay protocol can be found in the Supporting Information.

### Spectroscopic Measurements

To perform spectroscopic studies, both cell lines were seeded at low density (30,000 cells/well) on CaF_2_ optical windows (Crystran Ltd.) in 12-well cell culture plates (in duplicates). 24 h after seeding, cells were serum-starved for 2 h in a serum free medium and then treated with the selected CBD concentrations for 24 h or left untreated as control samples (0 μM CBD). Then, one set of samples per each cell line was irradiated with X-rays, as previously described. More details can be found in the Supporting Information.

### Raman Microspectroscopy

Single-cell Raman images were recorded using a Renishaw InVia Raman spectrometer equipped with an optical confocal microscope, an air-cooled solid-state laser emitting at 532 nm, and a CCD detector cooled to −70 °C. An immersive Olympus LUMPlanFL (60×, NA 1.0) objective was used.

### FT-IR Microspectroscopy

Single-cell FT-IR images were collected using a HYPERION 3000 FT-IR microscope with a 36× magnification objective, coupled with a Vertex 70v spectrometer (Bruker, Ettlingen, Germany) operating in the transmission mode. Hyperspectral images were recorded by the FPA detector of 64 × 64 pixels and projected pixel size 1.1 μm × 1.1 μm.

### AFM-IR Nanospectroscopy

AFM-IR spectra (10 cells per condition) and exemplary maps for selected cells were collected in the contact mode using the NanoIR2 spectrometer (Anasys Instrument, Santa Barbara, California) with silicon gold-coated PR-EX-nIR2 probes [30 nm tip diameter, 13 ± 4 kHz resonance frequency, (Anasys Instruments, USA)].

More details about the abovementioned systems and data analysis can be found in the Supporting Information.

## Results and Discussion

### Cyto- and Genotoxic Effect of CBD and CBD with the Combination of Ionizing Radiation

To investigate the CBD influence on Schwann and MPNST cell viability, various CBD concentrations were tested (Figure S4A, Supporting Information). Interestingly, after 24 h of incubation, increased viability of Schwann cells was observed, in the range of 0.01–5 μM CBD concentration, compared to untreated, control cells. Surprisingly, CBD treatment did not affect MPNST cell viability, despite its proven anticancer effect.^5,29^ For further investigations, 0.025, 0.1, 0.5, 3, and 9 μM concentrations were selected. Before the application of X-ray irradiation, the possible influence of CBD itself on DNA was estimated for both cell lines by comet assay. Based on electrophoresis and fluorescence staining, the observation of the damaged DNA as comet “tail” was possible, separated from the intact DNA “head”. Then, t-DNA_0Gy_ (tail DNA) values were calculated for both cell lines incubated with selected CBD concentrations, without irradiation procedure (0 Gy) (Figure S4B). The Pearson’s correlation coefficient statistically estimated the strength of a relationship between paired data, denoted by *r* and constrained as −1 ≤ *r* ≥ 1. *R* values were calculated from the polynomial fitting, and for t-DNA_0Gy_, they amount 0.48 and 0.51 for Schwann and MPNST cells, respectively. Both values were included in the 0.4–0.59 range (positive moderate correlation), what indicated relationship between the CBD concentrations and the level of DNA damage, although the indirect relationship was not strong. Therefore, CBD itself did not reveal any toxic effect on DNA for the Schwann and MPNST cell lines (Figure S4B).

On the contrary, the t-DNA_10Gy_ established for cells treated with CBD and irradiated with 10 Gy exposure dose displayed differences in the cell response to ionizing radiation in a dose-dependent manner (Figure S4C). For Schwann cell line, strong negative correlation appeared (−0.76 in the range −1 to −0.70), which designated that the higher CBD concentration, the lower DNA damage caused by ionizing radiation. Surprisingly, an opposing strong positive correlation (0.88 in the range from 0.7 to 1) was visible for MPNST, and therefore, the increase in the CBD concentration enhanced the toxic effect of radiotherapy, which was manifested in elevated DNA damage observed in the comet “tail”. Based on t-DNA_0Gy_ and t-DNA_10Gy_ values, it was possible to evaluate the relative susceptibility of cells to ionizing radiation. Radiosusceptibility was calculated as the difference in the levels of DNA damage immediately after (t-DNA_10Gy_) and before (t-DNA_0Gy_) radiation (Figure S4D). Remarkably, with an increased CBD concentration, a distinct cellular response to the 10 Gy exposure dose was observed. Both cell lines presented strong opposing correlation; however, for Schwann the negative value (−0.84) displayed the decrease in radiosusceptibility with an increased CBD concentration. For MPNST, high positive correlation (0.92) indicated the reverse trend, namely, strengthen in radiosusceptibility as the CBD concentration raised. Therefore, with an increased CBD concentration, under the influence of 10 Gy irradiation exposure dose, the DNA of Schwann cells was less damaged and become more resistant to ionizing radiation, whereas for MPNST, CBD enhanced DNA destruction and increased vulnerability to radiation.

### Spectroscopic Signature of CBD and CBD Combined with Radiation Influence on Schwann and MPNST Cells

Hyperspectral imaging, with the use of Raman and FT-IR spectroscopy, allows for simultaneous acquisition of biochemical information (spectra) with its distribution (chemical maps) in a micrometric scale, which can shed light on detailed biochemical changes occurring at the subcellular level. Because the purpose of this investigation was to determine the overall cell response to CBD and radiation, single-cell CA was used for selection mean Raman and FT-IR spectra representing each individual cell. Such an approach allows one to obtain precise information about the biochemical changes at the single-cell level.

**Figure 1:**
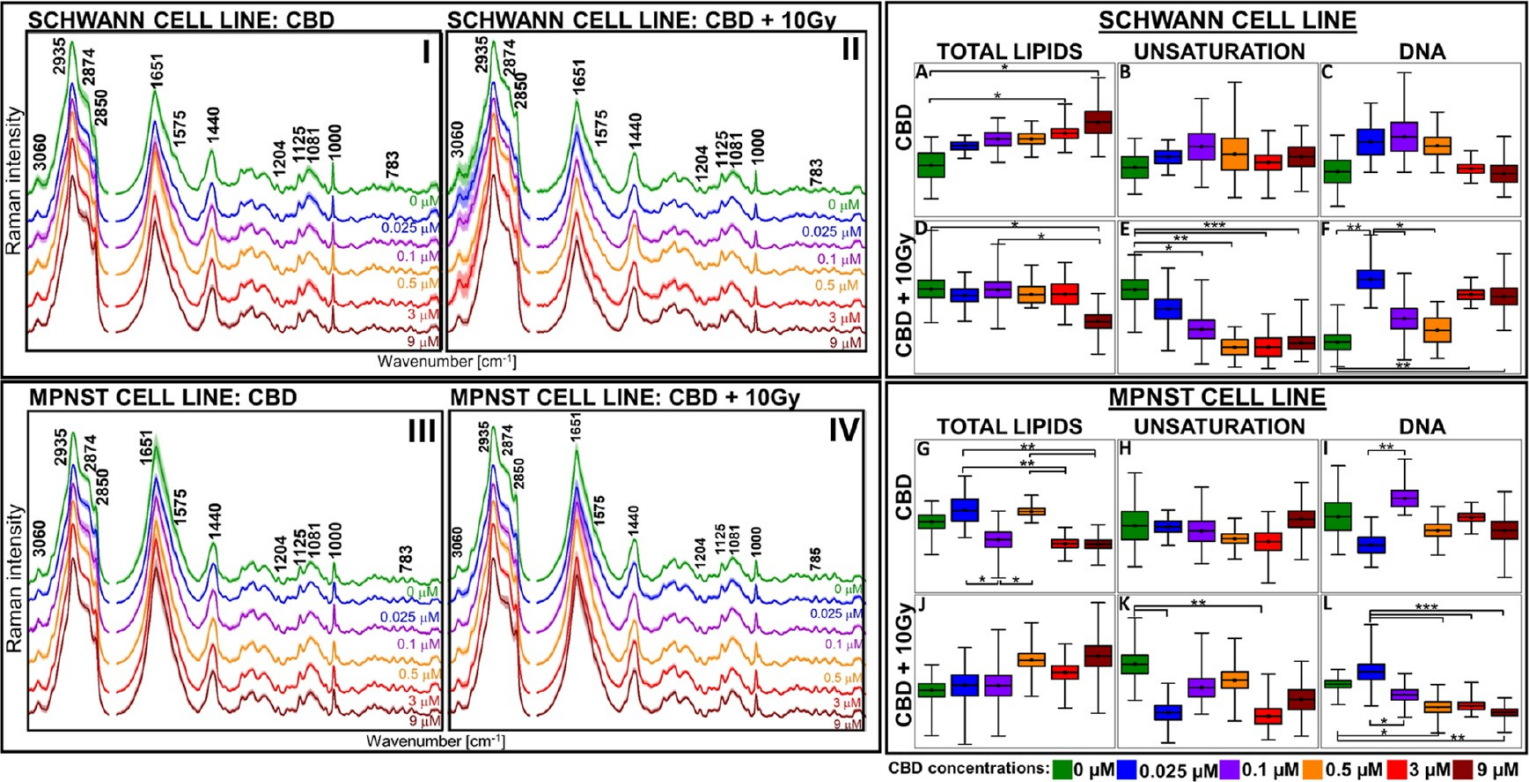
(I–IV) Correlation of averaged Raman spectra collected for Schwann (I,II) and MPNST (III,IV) cell lines incubated with CBD and CBD with 10 Gy X-ray exposure dose. Shading denotes standard deviation. The bands’ assignment is given in Table S1. (A–L) Box diagrams (mean with standard deviation and min–max range) of integral intensities for the selected Raman bands for Schwann and MPNST cells treated with CBD (A–C,G–I) and CBD with 10 Gy X-ray exposure dose (D–F,J–L). The integration ranges and bands’ assignment are given in the Supporting Information. Mean values of each presented box are given in Table S3 (Supporting Information).

**Figure 2:**
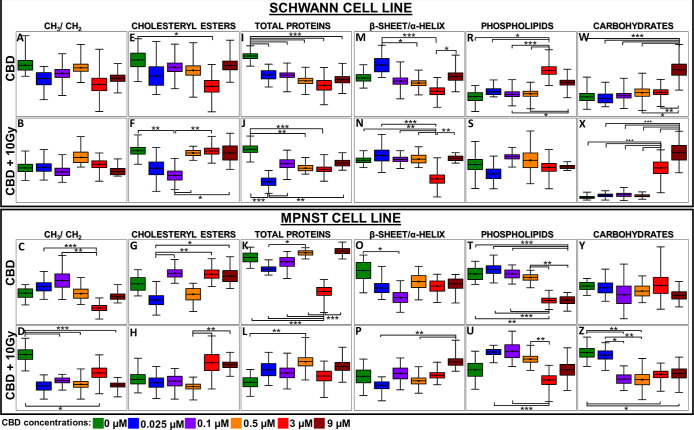
Comparison of box diagrams (mean with standard deviation and min–max range) of integral intensities for the selected FT-IR bands (A–Z) for Schwann (top panel) and MPNST (bottom panel) cell lines treated with CBD and CBD with 10 Gy X-ray exposure dose. The integration ranges and bands’ assignment are given in the Supporting Information. Mean values of each presented box is given in Table S4 (Supporting Information).

To emphasize biochemical changes caused by CBD and its combination with ionizing radiation, integral intensities of selected Raman and FT-IR bands were calculated (Figures 1 and 2). Also, to provide the outlook of the Raman and FT-IR spectral pattern for each experimental condition, single-cells spectra were averaged within a given experimental group afterward (Figures 1I–IV and 3A,B,E,F).

**Figure 3:**
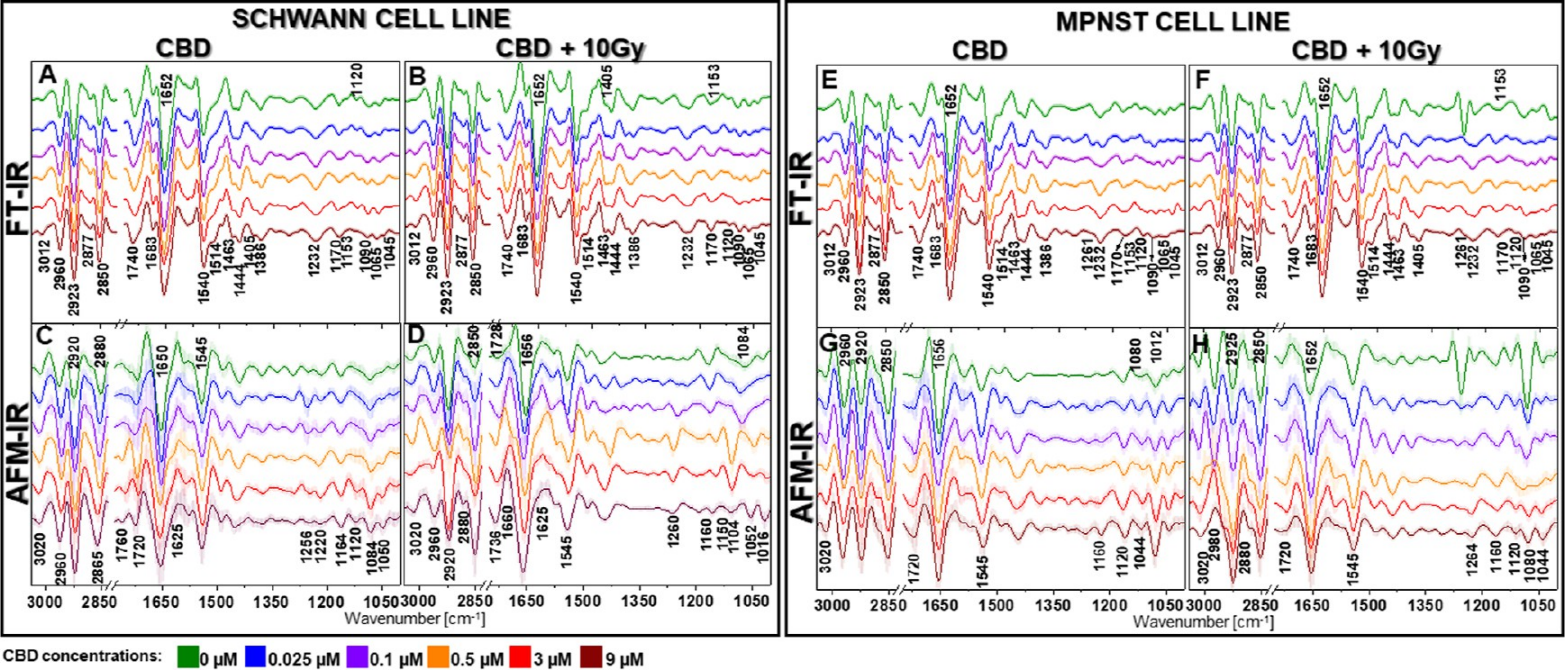
Correlation of mean second derivative spectra collected for Schwann and MPNST cell lines incubated with CBD and CBD with 10 Gy X-ray exposure dose: for conventional FT-IR (A,B,E,F) and AFM-IR system (C,D,G,H). Shading denotes standard deviation. The bands’ assignment is given in Table S2 (Supporting Information).

An increase in total lipid concentration for Schwann cells can be related to CBD indirect enhancement of the production of long-chain polyunsaturated fatty acids (FA) such as e.g., endocannabinoids, or increased lipid storage (Figure 1A).^30,31^ In the case of cancer cells, where lipid synthesis is usually elevated, CBD possibly inhibits this process (Figure 1G). Notably, the lipid unsaturation was not affected by CBD for both cell lines (Figure 1B,H).^32^ Any relevant change in the DNA of both cell lines after CBD treatment was observed (Figure 1C,I), what was consistent with the comet assay results for t-DNA_0Gy_. By contrast, after the implementation of CBD combined with radiation, for Schwann cells, the total amount of lipids significantly decreased and reached the lowest value for 9 μM CBD concentration, while in cancer cells, the opposing trend was observed but was not statistically relevant (Figure 1D,J). In case of lipid unsaturation, after radiation their amount diminished (Figure 1E,K) but for Schwann cells, this change was more gradual than that for MPNST. According to the literature, the lower level of lipids as well as their unsaturation may be associated with oxidation caused by ROS generated from ionizing radiation, inducing cell stress and lipid peroxidation.^33,34^ For MPNST, the enhanced content of lipids possibly relates to the stress therefore increased release from lipid droplets in order to possess energy for survival and repair.^35^ After irradiation, Schwann cells presented a significant increase in the nucleic acid concentration (DNA) for the 0.025, 3 and 9 μM CBD (Figure 1F). As CBD exhibit neuroprotective and antioxidant properties, it might reduce the concentration of free radicals and their harmful effects on DNA for normal cells.^36^ In contrast, the amount of DNA was strongly reduced for MPNST cells (Figure 1L) Therefore, with the higher CBD concentrations, the cancer cells become more vulnerable to radiation therapy; thus, CBD acts as a radiosensitizer and escalate the DNA damage as indicated by comet assay (Figure S4D).^37^

To possess profound information about protein transformation, modification in secondary protein structure, and cholesteryl esters or molecules as phospholipids and carbohydrates, FT-IR imaging was introduced. The integral intensity calculations for abovementioned molecules are presented as box-plots in Figure 2.

The ratio of CH_3_ to CH_2_ vibrations represents the alterations in the length of fatty acyl chains as well as their unsaturation.^38^ For Schwann cells, both CBD and the combination of CBD with X-ray did not affect the length of the fatty acyl chains (Figure 2A,B). For the same samples, Raman spectroscopy demonstrated the decrease in lipid unsaturation after the application of CBD and X-ray radiation (Figure 1E). Therefore, the length of fatty acid chains presented in Schwann cells did not change with CBD concentration but becomes more saturated and emerged as an important source of energy.^39^ The dissent observations were represented by MPNST, where the transformations in fatty acyl chains appeared only for 0.025, 0.1, and 3 μM CBD-treated cells; however, no significant correlation was present in contrast to untreated cells (Figure 2C). Based on Raman results, the lipid unsaturation remained consistent through each CBD concentration (Figure 1H) similarly as for Schwann cells (Figure 1B). Surprisingly, after MPNST irradiation, a strong decrease in the CH_3_/CH_2_ ratio was observed in these cells (Figure 2D). The predominance of –CH_2_ moieties was already mentioned in the literature for cancer cells after radiotherapy and interpreted as the apoptosis predicting factor.^40^ As an enhanced level of −CH_2_ groups correlates to apoptosis, such observation coincided with reduced DNA levels after irradiation (Figure 1L), thereby with increased radiosusceptibility (Figure S4D) for MPNST.

The cholesteryl esters were affected in both cell lines, notably for Schwann cells, and a significant diminish of this lipid fraction was observed only for 3 μM CBD concentration, in contrast to untreated cells (Figure 2E). It is known that CBD reduces the amount of FA, which are essential for cholesteryl ester synthesis.^41^ Therefore, in Schwann cells for 3 μM CBD concentrations, the level of FA might be limited and less accessible; hence, lower amounts of cholesteryl esters were synthesized.^42^ The introduction of irradiation led to a decrease in cholesteryl esters additionally for 0.1 μM CBD; however, for higher concentrations, this lipid fraction remained at the same level as for untreated cells. This distinct behavior of cholesteryl esters under the influence of CBD and X-ray treatment suggests different pathways of its metabolism for Schwann cells. CBD also influenced cholesteryl esters in cancer cells, although the trend of changes strongly depended on the CBD concentration and was not related to untreated cells (Figure 2G). After MPNST irradiation, the increased level of cholesteryl esters was more prominent for higher CBD concentrations (Figure 2H), as displayed for Schwann cells (Figure 2F). These might indicate that CBD increases the cholesterol biosynthesis, as well as cell storage for both, cholesterol and cholesteryl esters.^42,43^

The CBD treatment caused the total protein content to decrease gradually with an increased CBD concentration for Schwann cells (Figure 2I). This effect was partially preserved after X-ray treatment (Figure 2J). The opposing tendency was presented for cancer cells, where the protein amount reduced dramatically only for the 3 μM CBD concentration (Figure 2K). Moreover, CBD treatment combined with irradiation displayed a similar trend in the level of proteins as before irradiation; however, it was less manifested for MPNST (Figure 2K,L). Since CBD affects proteins mainly through protein inhibition or activation, the spectroscopic response may differ between Schwann and MPNST cell lines.^44^

It was also possible to investigate the modifications in terms of the secondary protein structures (Figure 2M–P). In Schwann cells, the 0.025 μM CBD directed the protein structure toward β-sheet, but as the CBD concentration increased, α-helical proteins began to predominate and display the greatest contribution for 3 μM CBD concentration. After irradiation, the presence of the β-sheet was no longer distinctive, yet for 3 μM the existence of the α-helix was still strongly manifested (Figure 2M,N). Usually, the appearance of β-sheet proteins is not desirable due to possible aggregation and transformation to amyloids as in neurodegenerative diseases or in cancer.^18,45^ Remarkably, higher CBD concentrations might modify the protein structure from β-sheet to α-helix or even suppress the formation of β-amyloids.^46^ For MPNST, lower CBD concentrations promoted the α-helical structures, while for 0.5–9 μM CBD concentrations, the ratio of β-sheet/α-helix persisted at the same level as for untreated cells. Importantly, after irradiation, the contribution of β-sheet structure increased gradually with CBD concentration and reached the highest value for 9 μM (Figure 2O,P).

The CBD influence on Schwann cells phospholipids was mainly observed for 3 and 9 μM CBD concentrations, whereas additional irradiation did not affect their content when compared to CBD-untreated cells (Figure 2R,S). Because CBD directly interacts with phospholipids, the increased production or storage of these lipid fractions in cells was observed.^47^ Interestingly, the additional X-ray treatment did not affect the level of phospholipids, and for each CBD concentration, it remained similar to that of CBD-untreated cells (Figure 3S). In turn, for cancer cells, at the same CBD concentrations (3 and 9 μM), the phospholipid content decreased significantly after treatment, opposing Schwann cells, and this trend persisted after irradiation (Figure 2T,U). Even though phospholipids were elevated in cancer cells’ membranes, due to disturbed metabolism, CBD reduced their amount and possibly facilitated easier cell damage with the ionizing radiation.^48,49^

CBD also induced changes in carbohydrate levels which occurred to be significantly increased for Schwann cells treated with 9 μM CBD. After the implementation of X-ray irradiation, additional rise in the amount of these molecules was displayed also for 3 μM (Figure 2W,X). In turn, CBD did not cause any changes in the cancer cells’ carbohydrates, but in combination with radiation, it significantly reduced their content even at a concentration as low as 0.1 μM (Figure 2Y,Z). Carbohydrates not only play an essential role as an energetic source but also in the recovery process.^50,51^ As observed for Schwann cell line, this process was more effective for higher CBD concentrations; nevertheless, in the case of cancer cells, it was disturbed and might contributed to increased radiosensitivity.

### Does Global FT-IR Information Correspond to the Local Infrared Nanospectroscopy Signature?

Conventional FT-IR imaging system allows one to assess rather bulk spectroscopic information due to its advantage in rapid scan collection, which provides general information from a statistical number of studied samples, with the offered spatial sampling of 1.1 μm. Unfortunately, such pixel size is not enough for deeper insights into alterations that may occur very locally in cells and are related to the spatial arrangement of biomolecules within cell morphology. Therefore, for a better understanding of modifications induced by CBD and radiation in Schwann and MPNST cells, AFM-IR nanospectroscopy was introduced. Due to the nanometer sizes of the AFM-IR tip, it was possible to collect spectra and chemical images with a spatial resolution of 40 nm.

AFM-IR second derivative spectra differed not only between the Schwann (Figure 3C,D) and MPNST (Figure 3G,H) cell lines but also varied within CBD concentrations. Therefore, AFM-IR reveals more detailed biochemical information than conventional FTIR, where the presented spectra were very similar (Figure 3A,B,E,F). For CBD-treated Schwann cells, AFM-IR disclosed significant changes in the bands’ intensity related to the cholesteryl esters (1760 cm^–1^) and FA (1720 cm^–1^) which suggested disturbed cholesteryl esters synthesis since FAs are their precursor (Figure 3C). Surprisingly, band characteristics for phosphates (1232 cm^–1^) split in two separate signals ca. 1256 and 1220 cm^–1^, which might indicate the changes in the conformation of the molecule-containing phosphates, e.g., in phospholipids (Figure 3C). Also, a band related to molecules with C–O groups (1120 cm^–1^), such as polysaccharides and ribose from RNA, was not present for 0 and 0.025 μM CBD concentrations; however, it increased gradually from 0.1 to 9 μM. Therefore, CBD impacted the ribose (RNA) and polysaccharides in a dose-dependent manner in Schwann cells. Conversely, conventional IR (Figure 3A) manifested the discussed band (1120 cm^–1^) for all CBD concentrations excluding only the highest 9 μM dose. Besides, fluctuation in the 1080 cm^–1^ (DNA) band intensity was more prominent for AFM-IR. In the case of irradiated Schwann cells, AFM-IR exhibited a significant shift from the 1728 cm^–1^ (untreated cells) to the 1736 cm^–1^ (9 μM CBD) band (Figure 3D). Therefore, CBD led to not only the alterations in cholesteryl esters amount according to previously shown box plots (Figure 2E–H) but also their structure. In contrast to non-irradiated Schwann cells, after irradiation, the spectral profile for 0.5 and 3 μM CBD concentrations displayed strong bands at 1260 and 1150 cm^–1^ and a significant shift from 1084 cm^–1^, related to phosphates in DNA and phospholipids, to 1104 cm^–1^, yet this trend disappeared for 9 μM CBD dose (Figure 3D). Described changes associated with DNA conformation might result in a radioresistance mechanism.

For cancer cells incubated with CBD, FT-IR showed similar lipid profiles between each condition (Figure 3E), while AFM-IR discriminated subsequent alterations in the 3020 and −2850 cm^–1^ lipid spectral range, mainly in the 2960 to 2920 cm^–1^ bands ratio. Interestingly, in contrast to FTIR (Figure 3E), AFM-IR did not reveal the presence of a 1683 cm^–1^ band, related to the β-sheet protein structure. This suggested that such protein conformation aggregates might be deposited globally within investigated cells, what was visible for overall FTIR cell screening. More prominent changes occurred in the 1160–1012 cm^–1^ spectral region, where the 1080 cm^–1^ band intensity increased gradually with CBD concentration (Figure 3G). This might relate to enhanced DNA activity. Since elevated production of DNA is not possible, we hypothesized that DNA was accumulated locally, therefore detected by AFM-IR.

After radiation, the most significant changes were visible for MPNST cells without CBD treatment, manifested by intense band at 1261 cm^–1^ observed for both FTIR and AFM-IR spectral profile (Figure 3F,H). For AFM-IR spectra, this band was more prominent and additionally accompanied by a sharp 1080 cm^–1^, which decreased significantly with the increased CBD concentration. This was possibly the result of DNA damage and decay resulting from ionizing radiation, the presence of CBD strikingly weakens X-ray toxicity; therefore, these bands were not observed for higher CBD concentrations.^52,53^ Also, the MPNST lipid profile changed dramatically, yet only for irradiated cells treated with CBD. Such alterations can be seen only by the AFM-IR technique as the 2980 cm^–1^ band shifts toward 2925 cm^–1^ and appeared as a shoulder for 3 μM CBD dose (Figure 3H).

On the contrary, the profile of the 2980–2850 cm^–1^ spectral range for the MPNST cells without CBD treatment displayed the same pattern as for untreated MPNST cells after irradiation (Figure 3G,H). Based on these observations, it can be noticed that the alterations in lipids presented for the cancer cells irradiated after CDB treatment were the result of the combination of CBD treatment with radiotherapy, hence not implicated from irradiation itself. Consequently, these modifications in lipids may play a key role in the increased radiosusceptibility of MPNST cells.

### AFM-IR Chemical Maps Reveal Changes in the Distribution of the Selected Molecules.

So far, the discussed Raman and FT-IR results allowed for the determination and semiquantitative analysis of the level of biochemical alterations generated by CBD or its combination with irradiation. Additionally, the AFM-IR technique provided information about the possible changes in the orientation^17,54−56^ or structure of DNA, cholesteryl esters, and phospholipids. Beside pronounced changes between the global Raman, FT-IR, and AFM-IR spectra profiles, also the significant differences in the spatial resolution of collected images were noticed. The imaging capabilities of those techniques are compared in Figure S5 (Supporting Information). The introduction of AFM-IR technique was necessary to gain knowledge about the modifications in local distribution of proteins (amide I, 1650 cm^–1^) and cholesteryl esters (1730 cm^–1^).

**Figure 4:**
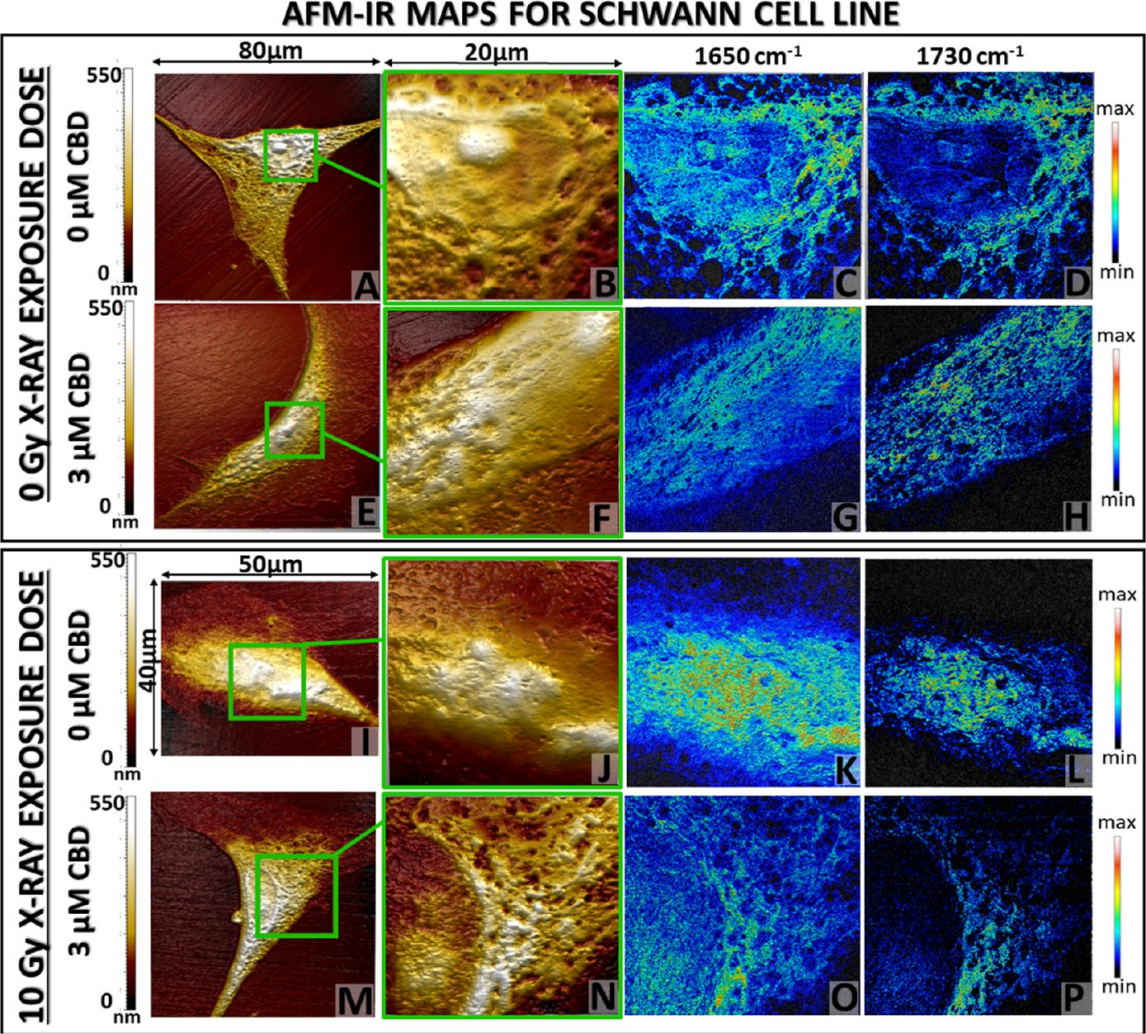
AFM topography of the whole Schwann cell (A,E,I,M) and selected area (green box: B,F,J,N) for nanoscale chemical mapping at 1650 cm^–1^ (C,G,K,O) and 1730 cm^–1^ (D,H,L,P) bands (scale bar: min 0 mV to max 200 mV). Collected images were selected for 3 μM CBD concentration in reference to the CBD untreated cells before (top) and after 10 Gy X-ray dose application (bottom).

**Figure 5:**
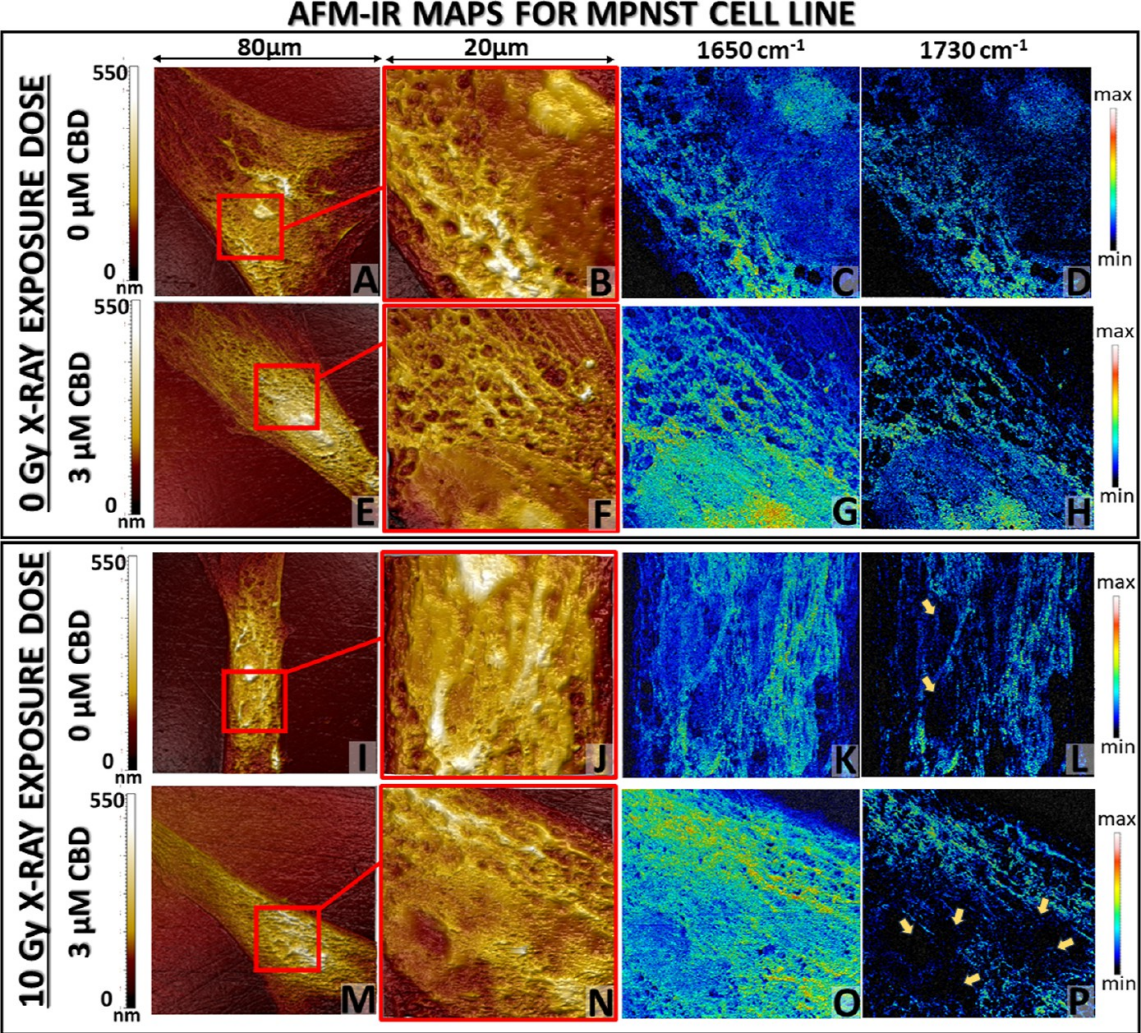
AFM topography of the whole MPNST cell (A,E,I,M) and selected area (red box: B,F,J,N) for nanoscale chemical mapping at 1650 cm^–1^ (C,G,K,O) and 1730 cm^–1^ (D,H,L,P) bands (scale bar: min 0 mV to max 200 mV). Collected images were selected for 3 μM CBD concentration in reference to the untreated cells without (top) or with 10 Gy X-ray dose application (bottom).

The exemplary AFM-IR maps (topography and chemical images) with the 40 nm spatial resolution were collected for Schwann (Figure 4) and MPNST (Figure 5) cells treated with 3 μM CBD concentration in contrast to control. The Schwann cell morphology without CBD treatment and irradiation displayed well-defined nuclei and nuclei, surrounded by cytoplasm containing pores (Figure 4A,B). An AFM-IR chemical map relates evenly distributed proteins within the whole cell area, in accordance with its morphology (Figure 4C,B). The signal originated from cholesteryl esters (1730 cm^–1^) displayed the strongest accumulation mainly around the nucleus, probably in the endoplasmic reticulum.^57^ After the incubation with a 3 μM concentration of CBD, the AFM topography map of the cell area (Figure 4E,F) presented also evident distribution of particular cellular structures such as nuclei subnuclear area; however, the margins between each element were less visible. The protein distribution colocalized with the signal from cholesteryl esters, but it was impossible to determine the accurate cell structure where it was accumulated, as was proposed for an untreated cell (Figure 4G,H). The irradiation of Schwann cells without CBD caused significant alterations in morphology (Figure 4I,J). The nuclei and cytoplasm presented no clear margins; therefore, it was difficult to determine their arrangement. Also, the protein signal was accumulated at the cell center, analogously to the cholesteryl ester distribution (Figure 4K,L). Surprisingly, the cell incubated with 3 μM CBD concentration and irradiated preserved the normal cell morphology (Figure 4M,N), with clearly differentiated nucleolus, nuclei, and its membrane as well as cytoplasm same as for nontreated and not irradiated Schwann cells (Figure 4A,B). Also, the chemical maps presented a more accurate distribution of proteins, representing cell area, and cholesteryl esters signal was gathered around the nuclei (Figure 4O,P).

AFM topography images of MPNST cells not exposed to X-ray radiation presented similar, well-defined morphology, and the addition of 3 μM CBD treatment did not affect their structure (Figure 5A,B,E,F). Both exhibited a clearly outlined nucleolus within the nucleus separated by a cytoplasmatic membrane. In the case of protein and cholesteryl ester distribution, the more intense signal was observed for cells treated with 3 μM CBD concentration, interestingly also in the nucleolus and nucleus area (Figure 5C,D,G,H). When cancer cells were exposed to ionizing radiation, significant changes in the cell morphology appeared. That manifested mainly in the nuclei deformation for both untreated and 3 μM CBD-treated MPNST cells even though the nucleolus was still differentiable in not treated cells (Figure 5I,J,M,N). The combination of 3 μM CBD with irradiation induced numerous hollows in the nuclei area and the disappearance of the nuclei membrane. For both, irradiated and non-irradiated cells, the protein signal was distributed within the whole cell area but was enhanced for 3 μM CBD treatment. The localization of cholesteryl esters was modified for both irradiated cells without and with CBD treatment. The 1730 cm^–1^ signal was scattered within the cell area and was absent in areas correlating to hollows presented by AFM topography (Figure 5L,P yellow arrows), which corresponded to the nuclei deformation.

The most significant alterations in the morphology and IR chemical maps were observed for Schwann and MPNST cells after radiation (Figures 4I–P and 5I–P). They proved that CBD treatment plays a key role in the modifications of cell morphology. For Schwann cells, despite the ionizing radiation, CBD treatment preserved normal morphology as well as protein and cholesteryl esters distribution, whereas for MPNST, CBD strengthened the ionizing radiation harmful effect which was manifested through the alterations in nuclei deformation and local disappearance of cholesteryl esters signal on chemoselective maps.

To confirm the observations resulting from AFM-IR mapping experiments, the disruption in the cholesteryl ester amount was also investigated by calculating the integral intensity of the 1730 cm^–1^ band for individual cells (*N* = 10 per condition, Figure S6, Supporting Information) from AFM-IR spectra (Figure 3). The amount of cholesteryl esters for Schwann cells after the implementation of 3 μM CBD concentration with irradiation remains at the similar level (mean: 7.107) as without any treatment (mean 7.276). However, for cancer cells, the cholesteryl ester level dramatically decreased when the combination of CBD and irradiation was applied (mean: 3.834) in comparison to untreated cancer cells (mean: 7.997) or cells irradiated (mean: 5.071) without the presence of CBD.

## Conclusions

**Figure 6:**
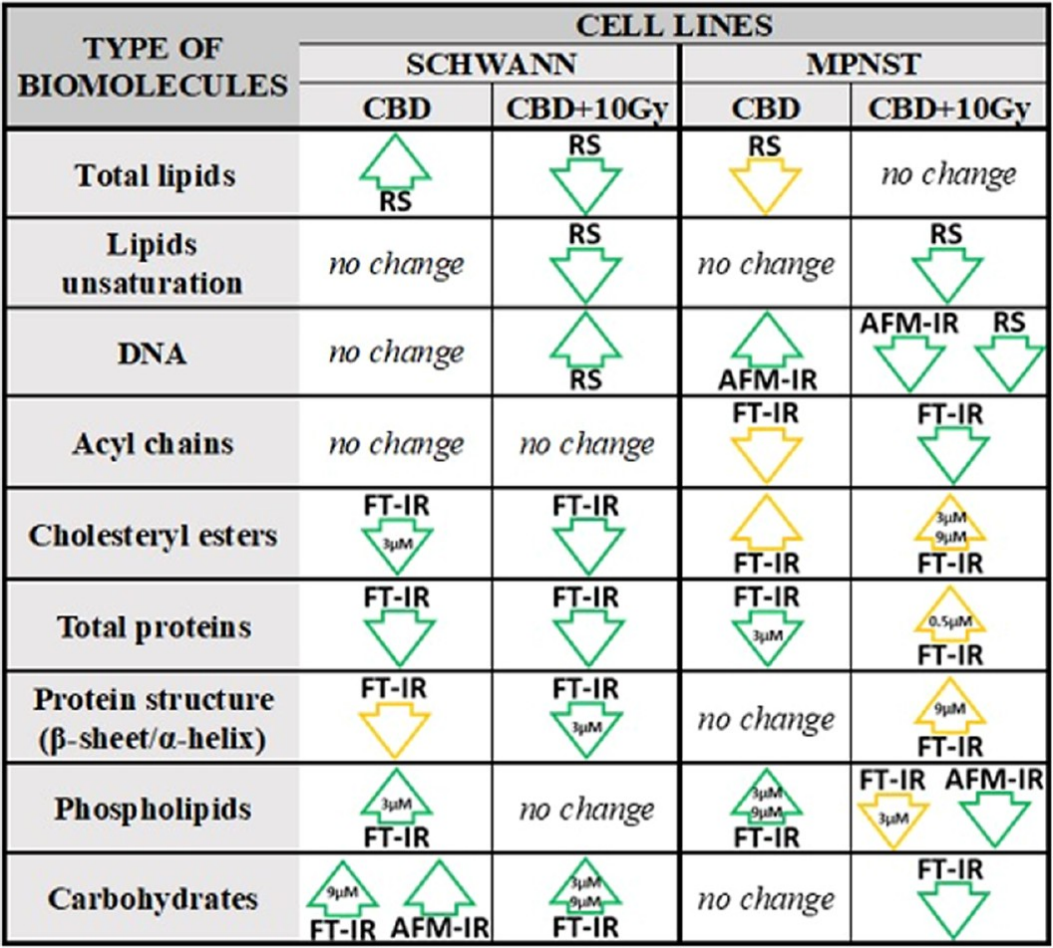
Summary of biochemical changes induced by CBD and CBD in combination with X-ray radiation on Schwann and MPNST cell lines. From Raman (RS), FT-IR, and AFM-IR data, it was possible to obtain information about the increase (arrow up) or decrease (arrow down) in the content of various types of biomolecules. Changes were statistically significant compared to the control (green contour) or between different CBD concentrations (yellow contour).

Performed studies indicated that the cells’ radiosusceptibility can be modified under the influence of CBD and its combination with X-ray radiation by alteration in various molecules, which has been summarized in Figure 6.

Raman spectroscopy was useful to determine the significant alterations in total lipids, which were increased after CBD treatment in Schwann cells but reduced in MPNST cells. Interestingly, their unsaturation decreases after irradiation for both cell lines. After irradiation, the level of Schwann cell’s DNA was elevated, whereas it decreases for MPNST. These fluctuations were accompanied by additional conformational changes in DNA structure for Schwann cells indicated by AFM-IR. FT-IR pointed out the shortening length of acyl chains which were characteristic for MPNST both treated with CBD and with the combination of CBD and irradiation. Cholesteryl esters exhibited distinct tendency, for Schwann cells their amount decreased while for MPNST their level was increased regardless of treatment combination; however, 3 μM CBD concentration was crucial to cholesteryl ester modifications.

Despite the application of CBD and its combination with irradiation, the level of proteins decreased along with predominance of α-helical structure with the greatest contribution for 3 μM CBD concentration after irradiation for Schwann cells. The secondary protein structure was also affected in irradiated MPNST, which was manifested by the dominance of β-sheet structure, especially for 9 μM CBD concentration. Phospholipids were mainly affected by 3 and 9 μM CBD concentrations, where for Schwann cells, their level rose along with the presence of conformational changes denoted by AFM-IR. Such observation was also presented for MPNST; however, after irradiation, this trend was reversed. Similar insights were exhibited for carbohydrates, which decreased only in the case of cancer cells treated with CBD and irradiation.

Based on those observations, it can be concluded that molecules that are involved in the cell response to X-ray radiation are DNA, cholesteryl esters, phospholipids, and carbohydrates. The use of hyperspectral imaging and the AFM-IR system allowed the broadening of knowledge in the field of biochemical modification introduced by CBD in the PNS in vitro model. With the combination of X-ray radiation and CBD, the toxicity of ionizing radiation in MPNST cells increases and simultaneously is reduced for Schwann cells.

## Supporting Information Available

Spectral characterization of CBD by Raman and FT-IR with band assignment and structural formula; cell cultivation with experimental workflow; MTS assay protocol; irradiation procedure, spectroscopic measurements, and data analysis; results of MTS assay for Schwann and MPNST cell lines; influence of selected CBD concentrations on Schwann and MPNST cells DNA indicated by comet assay; influence of selected CBD concentrations with combination of 10 Gy X-ray exposure dose on Schwann and MPNST cells DNA indicated by comet assay; calculation of the radiosusceptibility for Schwann and MPNST cells treated with selected CBD concentrations and irradiated with 10 Gy exposure dose; comparison of spatial resolution for FT-IR, Raman, and AFM-IR imaging techniques; calculations of integral intensities for 1730 cm^–1^ band related to cholesteryl esters based on the AFM-IR spectra; assignment of band position to biomolecules and vibrational modes for Raman and IR; and correlation of integral intensity mean values calculated for individual cells per experimental conditions (*N* = 15) for Raman and FT-IR data (PDF)

## References

[ref1] Mattiuzzi C.; Lippi G. Current Cancer Epidemiology. Clin. Epidemiol. Global Health 2019, 9 (4), 217 10.2991/jegh.k.191008.001. PMC7310786 31854162

[ref2] Debela D. T.; Muzazu S. G. Y.; Heraro K. D.; Ndalama M. T.; Mesele B. W.; Haile D. C.; Kitui S. K.; Manyazewal T. New Approaches and Procedures for Cancer Treatment: Current Perspectives. SAGE Open Med. 2021, 9, 205031212110343 10.1177/20503121211034366. PMC8366192 34408877

[ref3] Fouard O.; Daisne J. F.; Wanet M.; Regnier M.; Gustin T. Long-Term Volumetric Analysis of Vestibular Schwannomas Following Stereotactic Radiotherapy: Practical Implications for Follow-Up. Clin. Transl. Radiat. Oncol. 2022, 33, 1–6. 10.1016/j.ctro.2021.12.003. 34977365 PMC8688865

[ref4] Tsao M. N.; Sahgal A.; Xu W.; De Salles A.; Hayashi M.; Levivier M.; Phd L. M.; Martinez R.; Régis J.; Ryu S.; et al. Stereotactic Radiosurgery for Vestibular Schwannoma: International Stereotactic Radiosurgery Society (ISRS) Practice Guideline. J. Radiosurgery SBRT 2017, 5 (1), 5. PMC5675503 29296459

[ref5] Hinz B.; Ramer R. Cannabinoids as Anticancer Drugs: Current Status of Preclinical Research. Br. J. Cancer 2022, 127 (1), 1–13. 10.1038/s41416-022-01727-4. 35277658 PMC9276677

[ref6] Cásedas G.; Moliner C.; Maggi F.; Mazzara E.; López V. Evaluation of Two Different Cannabis Sativa L. Extracts as Antioxidant and Neuroprotective Agents. Front. Pharmacol. 2022, 13, 1009868 10.3389/fphar.2022.1009868. 36176449 PMC9513154

[ref7] Pagano S.; Coniglio M.; Valenti C.; Federici M. I.; Lombardo G.; Cianetti S.; Marinucci L. Biological Effects of Cannabidiol on Normal Human Healthy Cell Populations: Systematic Review of the Literature. Biomed. Pharmacother. 2020, 132, 110728 10.1016/j.biopha.2020.110728. 33038581

[ref8] Tripson M.; Litwa K.; Soderstrom K. Cannabidiol Inhibits Neuroinflammatory Responses and Circuit-Associated Synaptic Loss Following Damage to a Songbird Vocal Pre-Motor Cortical-like Region. Sci. Rep. 2023, 13 (1), 7907–7918. 10.1038/s41598-023-34924-z. 37193782 PMC10188539

[ref9] Deguchi M.; Potlakayala S.; Spuhler Z.; George H.; Sheri V.; Agili R.; Patel A.; Rudrabhatla S. Selection and Validation of Reference Genes for Normalization of QRT-PCR Data to Study the Cannabinoid Pathway Genes in Industrial Hemp. PLoS One 2021, 16 (12), e0260660 10.1371/JOURNAL.PONE.0260660. 34928958 PMC8687539

[ref10] Chrabąszcz K.; Kołodziej M.; Roman M.; Pięta E.; Piergies N.; Rudnicka-Czerwiec J.; Bartosik-Psujek H.; Paluszkiewicz C.; Cholewa M.; Kwiatek W. M. Carotenoids Contribution in Rapid Diagnosis of Multiple Sclerosis by Raman Spectroscopy. Biochim. Biophys. Acta, Gen. Subj. 2023, 1867 (9), 130395 10.1016/j.bbagen.2023.130395. 37271406

[ref11] Blat A.; Dybas J.; Kaczmarska M.; Chrabaszcz K.; Bulat K.; Kostogrys R. B.; Cernescu A.; Malek K.; Marzec K. M. An Analysis of Isolated and Intact Rbc Membranes - a Comparison of a Semiquantitative Approach by Means of FTIR, Nano-FTIR, and Raman Spectroscopies. Anal. Chem. 2019, 91 (15), 9867–9874. 10.1021/acs.analchem.9b01536. 31241915

[ref12] Notarstefano V.; Sabbatini S.; Conti C.; Pisani M.; Astolfi P.; Pro C.; Rubini C.; Vaccari L.; Giorgini E. Investigation of Human Pancreatic Cancer Tissues by Fourier Transform Infrared Hyperspectral Imaging. J. Biophotonics 2020, 13 (4), e201960071 10.1002/jbio.201960071. 31648419

[ref13] Chrabaszcz K.; Jasztal A.; Smęda M.; Zieliński B.; Blat A.; Diem M.; Chlopicki S.; Malek K.; Marzec K. M. Label-Free FTIR Spectroscopy Detects and Visualizes the Early Stage of Pulmonary Micrometastasis Seeded from Breast Carcinoma. Biochim. Biophys. Acta, Mol. Basis Dis. 2018, 1864 (11), 3574–3584. 10.1016/j.bbadis.2018.08.022. 30251677

[ref14] Chrabaszcz K.; Meyer T.; Bae H.; Schmitt M.; Jasztal A.; Smeda M.; Stojak M.; Popp J.; Malek K.; Marzec K. M. Comparison of Standard and HD FT-IR with Multimodal CARS/TPEF/SHG/FLIMS Imaging in the Detection of the Early Stage of Pulmonary Metastasis of Murine Breast Cancer. Analyst 2020, 145, 4982–4990. 10.1039/d0an00762e. 32515437

[ref15] Deng X.; Ali-Adeeb R.; Andrews J. L.; Shreeves P.; Lum J. J.; Brolo A.; Jirasek A. Monitor Ionizing Radiation-Induced Cellular Responses with Raman Spectroscopy, Non-Negative Matrix Factorization, and Non-Negative Least Squares. Appl. Spectrosc. 2020, 74, 701–711. 10.1177/0003702820906221. 32098482

[ref16] Roman M.; Wrobel T. P.; Paluszkiewicz C.; Kwiatek W. M. Comparison between High Definition FT-IR, Raman and AFM-IR for Subcellular Chemical Imaging of Cholesteryl Esters in Prostate Cancer Cells. J. Biophotonics 2020, 13 (5), e201960094 10.1002/jbio.201960094. 31999078

[ref17] Dazzi A.; Prater C. B.; Hu Q.; Chase D. B.; Rabolt J. F.; Marcott C. AFM–IR: Combining Atomic Force Microscopy and Infrared Spectroscopy for Nanoscale Chemical Characterization. Appl. Spectrosc. 2012, 66 (12), 1365–1384. 10.1366/12-06804. 23231899

[ref18] Paluszkiewicz C.; Piergies N.; Guidi M. C.; Pięta E.; Ścierski W.; Misiołek M.; Drozdzowska B.; Ziora P.; Lisowska G.; Kwiatek W. M. Nanoscale Infrared Probing of Amyloid Formation within the Pleomorphic Adenoma Tissue. Biochim. Biophys. Acta, Gen. Subj. 2020, 1864 (10), 129677 10.1016/j.bbagen.2020.129677. 32634535

[ref19] Paluszkiewicz C.; Piergies N.; Chaniecki P.; Rękas M.; Miszczyk J.; Kwiatek W. M. Differentiation of Protein Secondary Structure in Clear and Opaque Human Lenses: AFM – IR Studies. J. Pharm. Biomed. Anal. 2017, 139, 125–132. 10.1016/j.jpba.2017.03.001. 28279927

[ref20] Ruggeri F. S.; Mannini B.; Schmid R.; Vendruscolo M.; Knowles T. P. J. Single molecule secondary structure determination of proteins through infrared absorption nanospectroscopy. Nanospectroscopy 2020, 11, 2945 10.1038/s41467-020-16728-1. PMC7287102 32522983

[ref21] Piergies N.; Mathurin J.; Dazzi A.; Deniset-Besseau A.; Oćwieja M.; Paluszkiewicz C.; Kwiatek W. M. IR Nanospectroscopy to Decipher Drug/Metal Nanoparticle Interactions: Towards a Better Understanding of the Spectral Signal Enhancement and Its Distribution. Appl. Surf. Sci. 2023, 609, 155217 10.1016/j.apsusc.2022.155217.

[ref22] Mathurin J.; Deniset-Besseau A.; Bazin D.; Dartois E.; Wagner M.; Dazzi A. Photothermal AFM-IR Spectroscopy and Imaging: Status, Challenges, and Trends. J. Appl. Phys. 2022, 131 (1), 010901 10.1063/5.0063902.

[ref23] Schwartz J. J.; Jakob D. S.; Centrone A. A Guide to Nanoscale IR Spectroscopy: Resonance Enhanced Transduction in Contact and Tapping Mode AFM-IR. Chem. Soc. Rev. 2022, 51 (13), 5248–5267. 10.1039/D2CS00095D. 35616225

[ref24] Surmacki J. M.; Woodhams B. J.; Haslehurst A.; Ponder B. A. J.; Bohndiek S. E. Raman Micro-Spectroscopy for Accurate Identification of Primary Human Bronchial Epithelial Cells. Sci. Rep. 2018, 8 (1), 12604–12611. 10.1038/s41598-018-30407-8. 30135442 PMC6105656

[ref25] Czamara K.; Majzner K.; Selmi A.; Baranska M.; Ozaki Y.; Kaczor A. Unsaturated Lipid Bodies as a Hallmark of Inflammation Studied by Raman 2D and 3D Microscopy. Sci. Rep. 2017, 7, 40889 10.1038/srep40889. 28098251 PMC5241649

[ref26] Kołodziej M.; Chrabąszcz K.; Pięta E.; Piergies N.; Rudnicka-Czerwiec J.; Bartosik-Psujek H.; Paluszkiewicz C.; Cholewa M.; Kwiatek W. M. Spectral Signature of Multiple Sclerosis. Preliminary Studies of Blood Fraction by ATR FTIR Technique. Biochem. Biophys. Res. Commun. 2022, 593, 40–45. 10.1016/j.bbrc.2022.01.046. 35051781

[ref27] Pięta E.; Chrabąszcz K.; Pogoda K.; Suchy K.; Paluszkiewicz C.; Kwiatek W. M. Adaptogenic Activity of Withaferin A on Human Cervical Carcinoma Cells Using High-Definition Vibrational Spectroscopic Imaging. Biochim. Biophys. Acta, Mol. Basis Dis. 2023, 1869 (2), 166615 10.1016/j.bbadis.2022.166615. 36481485

[ref28] Chrabaszcz K.; Pogoda K.; Ciezak K.; Panek A.; Kwiatek W. M. High Resolution Optical Spectroscopy for the Evaluation of Cannabidiol Efficiency as a Radiation Therapy Support of Peripheral Nervous System Tumors. 2023, bioRxiv 2023.12.11.571087.

[ref29] O’Reilly E.; Khalifa K.; Cosgrave J.; Azam H.; Prencipe M.; Simpson J. C.; Gallagher W. M.; Perry A. S. Cannabidiol Inhibits the Proliferation and Invasiveness of Prostate Cancer Cells. J. Nat. Prod. 2023, 86, 2151–2161. 10.1021/acs.jnatprod.3c00363. 37703852 PMC10521019

[ref30] Chiocchetti R.; Galiazzo G.; Tagliavia C.; Stanzani A.; Giancola F.; Menchetti M.; Militerno G.; Bernardini C.; Forni M.; Mandrioli L. Cellular Distribution of Canonical and Putative Cannabinoid Receptors in Canine Cervical Dorsal Root Ganglia. Front. Vet. Sci. 2019, 6, 313 10.3389/fvets.2019.00313. 31608295 PMC6761858

[ref31] Chang R. C.; Thangavelu C. S.; Joloya E. M.; Kuo A.; Li Z.; Blumberg B. Cannabidiol Promotes Adipogenesis of Human and Mouse Mesenchymal Stem Cells via PPARγ by Inducing Lipogenesis but Not Lipolysis. Biochem. Pharmacol. 2022, 197, 114910 10.1016/j.bcp.2022.114910. 35026188 PMC8917779

[ref32] Butler L. M.; Perone Y.; Dehairs J.; Lupien L. E.; de Laat V.; Talebi A.; Loda M.; Kinlaw W. B.; Swinnen J. V. Lipids and Cancer: Emerging Roles in Pathogenesis, Diagnosis and Therapeutic Intervention. Adv. Drug Delivery Rev. 2020, 159, 245–293. 10.1016/j.addr.2020.07.013. PMC7736102 32711004

[ref33] Ye L. F.; Chaudhary K. R.; Zandkarimi F.; Harken A. D.; Kinslow C. J.; Upadhyayula P. S.; Dovas A.; Higgins D. M.; Tan H.; Zhang Y.; et al. Radiation-Induced Lipid Peroxidation Triggers Ferroptosis and Synergizes with Ferroptosis Inducers. ACS Chem. Biol. 2020, 15 (2), 469–484. 10.1021/acschembio.9b00939. 31899616 PMC7180072

[ref34] Yuan Z. H.; Liu T.; Wang H.; Xue L. X.; Wang J. J. Fatty Acids Metabolism: The Bridge Between Ferroptosis and Ionizing Radiation. Front. Cell Dev. Biol. 2021, 9, 1–15. 10.3389/fcell.2021.675617. PMC8264768 34249928

[ref35] Koizume S.; Miyagi Y. Lipid Droplets: A Key Cellular Organelle Associated with Cancer Cell Survival under Normoxia and Hypoxia. Int. J. Mol. Sci. 2016, 17 (9), 1430 10.3390/ijms17091430. 27589734 PMC5037709

[ref36] Smith T. A.; Kirkpatrick D. R.; Smith S.; Smith T. K.; Pearson T.; Kailasam A.; Herrmann K. Z.; Schubert J.; Agrawal D. K. Radioprotective Agents to Prevent Cellular Damage Due to Ionizing Radiation. J. Transl. Med. 2017, 15 (1), 232 10.1186/s12967-017-1338-x. 29121966 PMC5680756

[ref37] Gong L.; Zhang Y.; Liu C.; Zhang M.; Han S. Application of Radiosensitizers in Cancer Radiotherapy. Int. J. Nanomedicine 2021, 16, 1083–1102. 10.2147/IJN.S290438. 33603370 PMC7886779

[ref38] Staniszewska-Slezak E.; Wiercigroch E.; Fedorowicz A.; Buczek E.; Mateuszuk L.; Baranska M.; Chlopicki S.; Malek K. A Possible Fourier Transform Infrared-Based Plasma Fingerprint of Angiotensin-Converting Enzyme Inhibitor-Induced Reversal of Endothelial Dysfunction in Diabetic Mice. J. Biophotonics 2018, 11, 1–11. 10.1002/jbio.201700044. 28700133

[ref39] Salsinha A. S.; Socodato R.; Relvas J. B.; Pintado M. The Pro- and Anti-inflammatory Activity of Fatty Acids. In Bioactive Lipids; Pintado M., Machado M., Gomes A. M., Salsinha A. S., Rodríguez-Alcalá L. M., Eds.; Academic Press, 2023; pp 51−75. 10.1016/B978-0-12-824043-4.00002-6

[ref40] Read G. H.; Bailleul J.; Vlashi E.; Kesarwala A. H. Metabolic Response to Radiation Therapy in Cancer. Mol. Carcinog. 2022, 61 (2), 200–224. 10.1002/mc.23379. 34961986 PMC10187995

[ref41] Bielawiec P.; Dziemitko S.; Konstantynowicz-Nowicka K.; Chabowski A.; Dzięcioł J.; Harasim-Symbor E. Cannabidiol Improves Muscular Lipid Profile by Affecting the Expression of Fatty Acid Transporters and Inhibiting de Novo Lipogenesis. Sci. Rep. 2023, 13 (1), 3694–3716. 10.1038/s41598-023-30872-w. 36879113 PMC9988888

[ref42] Guard S. E.; Chapnick D. A.; Poss Z. C.; Ebmeier C. C.; Jacobsen J.; Nemkov T.; Ball K. A.; Webb K. J.; Simpson H. L.; Coleman S.; et al. Multiomic Analysis Reveals Disruption of Cholesterol Homeostasis by Cannabidiol in Human Cell Lines. Mol. Cell. Proteomics 2022, 21 (10), 100262 10.1016/j.mcpro.2022.100262. 35753663 PMC9525918

[ref43] Rimmerman N.; Juknat A.; Kozela E.; Levy R.; Bradshaw H. B.; Vogel Z. The Non-Psychoactive Plant Cannabinoid, Cannabidiol Affects Cholesterol Metabolism-Related Genes in Microglial Cells. Cell. Mol. Neurobiol. 2011, 31 (6), 921–930. 10.1007/s10571-011-9692-3. 21533611 PMC11498456

[ref44] Abyadeh M.; Gupta V.; Paulo J. A.; Gupta V.; Chitranshi N.; Godinez A.; Saks D.; Hasan M.; Amirkhani A.; McKay M.; et al. A Proteomic View of Cellular and Molecular Effects of Cannabis. Biomolecules 2021, 11 (10), 1411 10.3390/biom11101411. 34680044 PMC8533448

[ref45] Rukmangadachar L. A.; Bollu P. C. Amyloid Beta Peptide; StatPearls, 2023; Vol. 3–5. 29083757

[ref46] Alali S.; Riazi G.; Ashrafi-Kooshk M. R.; Meknatkhah S.; Ahmadian S.; Hooshyari Ardakani M.; Hosseinkhani B. Cannabidiol Inhibits Tau Aggregation In Vitro. Cells 2021, 10 (12), 3521 10.3390/CELLS10123521. 34944028 PMC8700709

[ref47] Atalay S.; Jarocka-karpowicz I.; Skrzydlewska E. Antioxidative and Anti-Inflammatory Properties of Cannabidiol. Antioxidants 2019, 9 (1), 21 10.3390/antiox9010021. 31881765 PMC7023045

[ref48] Cheng M.; Bhujwalla Z. M.; Glunde K. Targeting Phospholipid Metabolism in Cancer. Front. Oncol. 2016, 6, 266 10.3389/fonc.2016.00266. 28083512 PMC5187387

[ref49] Van Der Paal J.; Neyts E. C.; Verlackt C. C. W.; Bogaerts A. Effect of Lipid Peroxidation on Membrane Permeability of Cancer and Normal Cells Subjected to Oxidative Stress. Chem. Sci. 2016, 7 (1), 489–498. 10.1039/C5SC02311D. 28791102 PMC5518669

[ref50] Chen Y.; Pan Y.; Feng Y.; Li D.; Man J.; Feng L.; Zhang D.; Chen H.; Chen H. Role of Glucose in the Repair of Cell Membrane Damage during Squeeze Distortion of Erythrocytes in Microfluidic Capillaries. Lab Chip 2021, 21 (5), 896–903. 10.1039/D0LC00411A. 33432946

[ref51] Holesh J. E.; Aslam S.; Martin A. Physiology, Carbohydrates. StatPearls, 2023. 29083823

[ref52] Borrego-Soto G.; Ortiz-López R.; Rojas-Martínez A. Ionizing Radiation-Induced DNA Injury and Damage Detection in patientswith Breast Cancer. Genet. Mol. Biol. 2015, 38 (4), 420–432. 10.1590/S1415-475738420150019. 26692152 PMC4763322

[ref53] Reisz J. A.; Bansal N.; Qian J.; Zhao W.; Furdui C. M. Effects of Ionizing Radiation on Biological Molecules—Mechanisms of Damage and Emerging Methods of Detection. Antioxid. Redox Signal. 2014, 21 (2), 260–292. 10.1089/ars.2013.5489. 24382094 PMC4060780

[ref54] Giliberti V.; Badioli M.; Nucara A.; Calvani P.; Ritter E.; Puskar L.; Aziz E. F.; Hegemann P.; Schade U.; Ortolani M.; Baldassarre L. Heterogeneity of the Transmembrane Protein Conformation in Purple Membranes Identified by Infrared Nanospectroscopy. Small 2017, 13 (44), 01181 10.1002/smll.201701181. 28960799

[ref55] Lu F.; Jin M.; Belkin M. A. Tip-Enhanced Infrared Nanospectroscopy via Molecular Expansion Force Detection. Nat. Photonics 2014, 8 (4), 307–312. 10.1038/nphoton.2013.373.

[ref56] Stöckle R. M.; Suh Y. D.; Deckert V.; Zenobi R. Nanoscale Chemical Analysis by Tip-Enhanced Raman Spectroscopy. Chem. Phys. Lett. 2000, 318 (1–3), 131–136. 10.1016/S0009-2614(99)01451-7.

[ref57] Kennelly J. P.; Tontonoz P. Cholesterol Transport to the Endoplasmic Reticulum. Cold Spring Harb. Perspect. Biol. 2023, 15 (2), a041263 10.1101/CSHPERSPECT.A041263. 35940908 PMC9899650

