# Supplementary material for: Sensing Biomolecules Associated with Cells’ Radiosusceptibility by Advanced Micro- and Nanospectroscopy Techniques
Source: ACS Sens. 2024 Sep 18;9(9):4887–97. doi: 10.1021/acssensors.4c01455 (PMC11443521; doi:10.1021/acssensors.4c01455)
Supplement: Supplementary file 1 — se4c01455_si_001.pdf [file se4c01455_si_001.pdf]

Supporting Information

Sensing biomolecules associated with cells' radiosusceptibility by advanced micro- and nanospectroscopy techniques

Karolina Chrabąszcz*, Katarzyna Pogoda, Klaudia Cieżak, Agnieszka Panek and Wojciech M. Kwiatek

Institute of Nuclear Physics Polish Academy of Sciences, Radzikowskiego 152, 31-342 Krakow, Poland
*corresponding author e-mail: karolina.chrabaszcz@ifj.edu.pl

KEYWORDS: spectroscopic detection; radiosusceptibility; microspectroscopy; nanospectroscopy; atomic force microscopy

Spectral characterization of cannabidiol:

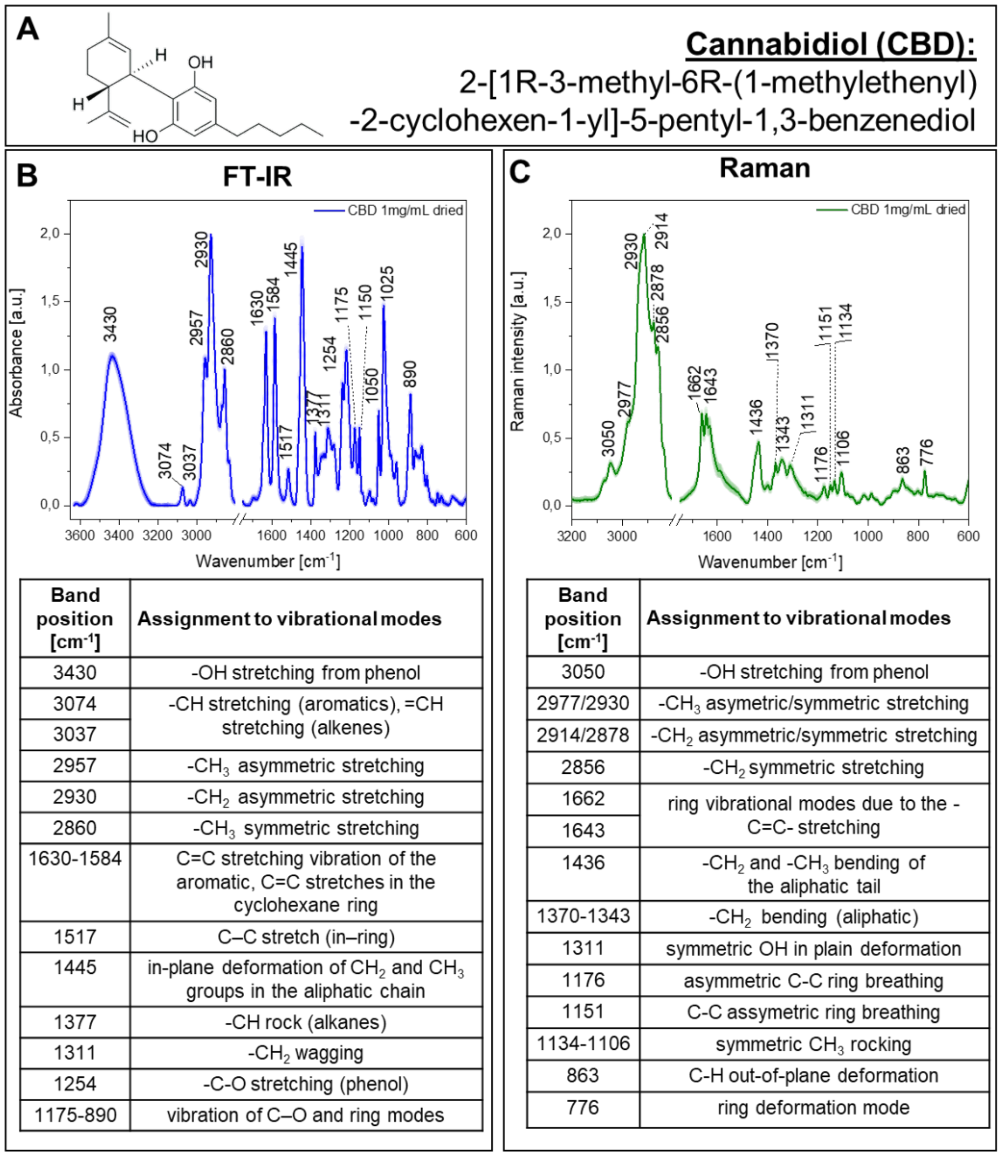

Band position [cm-1]	Assignment to vibrational modes
3430	-OH stretching from phenol
3074	-CH stretching (aromatics), =CH stretching (alkenes)
3037	
2957	$-CH_3$ asymmetric stretching
2930	$-CH_2$ asymmetric stretching
2860	$-CH_3$ symmetric stretching
1630-1584	C=C stretching vibration of the aromatic, C=C stretches in the cyclohexane ring
1517	C–C stretch (in–ring)
1445	in-plane deformation of CH_2 and CH_3 groups in the aliphatic chain
1377	-CH rock (alkanes)
1311	$-CH_2$ wagging
1254	-C-O stretching (phenol)
1175-890	vibration of C–O and ring modes

Band position [cm-1]	Assignment to vibrational modes
3050	-OH stretching from phenol
2977/2930	$-CH_3$ asymetric/symmetric stretching
2914/2878	$-CH_2$ asymmetric/symmetric stretching
2856	$-CH_2$ symmetric stretching
1662	ring vibrational modes due to the -C=C- stretching
1643	
1436	$-CH_2$ and $-CH_3$ bending of the aliphatic tail
1370-1343	$-CH_2$ bending (aliphatic)
1311	symmetric OH in plain deformation
1176	asymmetric C-C ring breathing
1151	C-C assymetric ring breathing
1134-1106	symmetric CH_3 rocking
863	C-H out-of-plane deformation
776	ring deformation mode

Figure S1. (A) The structural formula of cannabidiol. Averaged spectra of cannabidiol measured from the dried drop of the initial solution used in the experiment (1mg/mL CBD in methanol); (B) averaged FT-IR spectrum of

cannabidiol collected from 20 points in reflection mode with bands assignment; (C) averaged Raman spectrum of cannabidiol collected from 20 points with bands assignment. Shading denotes standard deviation. Bands assignments were made according to the literature.[1-5]

Materials and methods:

Cell culture: Cells were cultured according to ATCC protocol in DMEM medium, supplemented with 10% of FBS (fetal bovine serum), 100 U/ml penicillin–streptomycin-neomycin solution. MPNST cell line was additionally supplemented with L-glutamine. The cells were grown in 75 cm^3 flasks at 37°C in an atmosphere of 5% CO_2. For spectroscopic studies cells were seeded on calcium fluoride windows (Crystran Ltd., UK) inside 12-well plates and kept in an incubator at 5% CO_2 and 37°C for 24 h to promote adhesion and growth. Scheme of experimental wokflow is present in Figure S2.

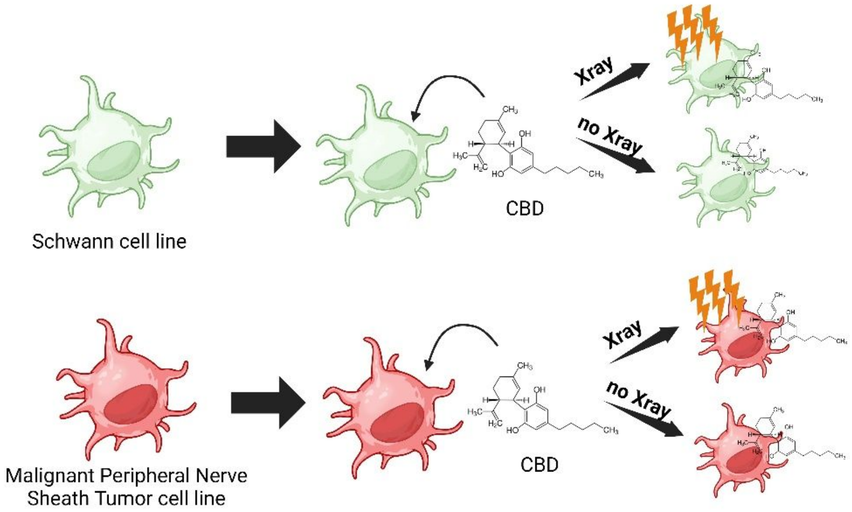

Figure S2. Scheme of experimental workflow.

MTS assay: Cells were seeded in 24-well plates at a density of 15, 000 /well and 24h after seeding cells were serum-starved for 2h in DPBD with Ca^{2+} and Mg^{2+}. Next, cells were treated with a selected concentration of CBD. Since the MTS assay is performed on live cells, particular MTS read-outs after 24h, 48h, and 72h of incubation with CBD were carried out on a single 24-well plate, and not at three separate plates, for every time point (Figure S3). CellTiter 96® AQueous One Solution reagent was added directly to the culture wells and incubated for 1 hour. Then the absorbance of formazan was recorded at 490 nm with a Spark 10 M (Tecan) multimode microplate reader. The absorbance values obtained with the untreated cells (control) were used for data normalization. Assays were conducted in triplicate. For spectroscopic measurements 24h time point was selected.

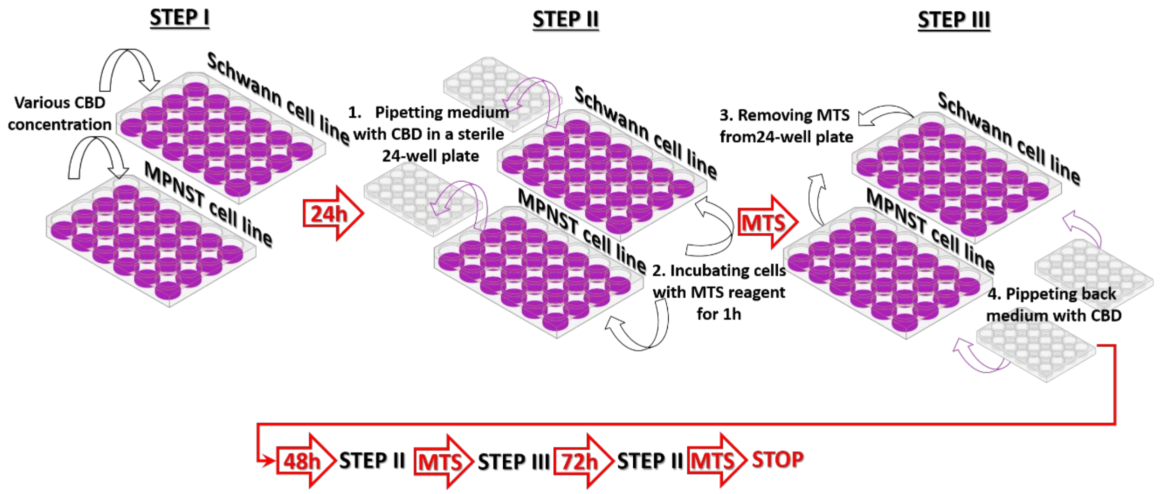

Figure S3. Schematic workflow of MTS assay cytotoxicity assay.

__**Irradiation procedure:**__ For this experiment, 10Gy exposure dose was selected as commonly used for MPNST radiotherapy. To maintain the same experimental condition, the set of non-irradiated samples were kept out of the incubator (Figure S2, *no Xray*) during the irradiation of the second set of samples (Figure S2, *Xray*).

__**Comet assay**__ Suspension (50 µl) of Schwann and MPNST cells (3000-4000 per 1 ml) have been embedded in 150 µl of agarose on a microscope slide. Cells were lysed for 1h by 1% Triton X-100 in pH> 13. Alkaline electrophoresis (30 V, and 300 mA) was then carried out for 30 min at 4°C. Ethidium bromide (17 mg/ml) was used for cells staining. Epifluorescence microscope Olympus BX-50 connected with a CCD camera (excitation filter 515–560 nm, barrier filter from 590 nm) was used for cellular DNA visualization. Semi-automated image analysis system Komet 3.0 software (Kinetic Imaging Co., Liverpool, UK) was applied for the analysis. The extent of DNA damage was determined by the T-DNA parameter – tail DNA (DNA percentage in the comet tail). Two independent experimental replicates were performed for each aliquot: from 200 to 500 cells were analyzed for each data point (2 slides per each CBD concentration, without and with 10Gy X-ray dose, 100–250 cells from each slide). Pearson's r correlation coefficient for data presented from comet assay were calculated from polynomial fitting.

__**Spectroscopic measurements:**__

For further studies, samples were rinsed twice with PBS for 5 min and fixing with 4% paraformaldehyde (PFA) in phosphate-buffered saline (PBS) for 20 min. Then, all samples were washed with PBS (3 times for 2 min) to remove PFA residues. For Raman measurements, samples were left in PBS solution. Before FT-IR and AFM-IR measurements samples were washed in a series PBS/water solutions and ultrapure water (9:1, 8:2, 7:3; 6:4, 5:5, 4:6, 3:7, 2:8, 1:9, for 2 min) then dried under a gentle stream of nitrogen. All solutions were prepared using ultrapure water (Di-rect-Q 3 UV, Millipore, USA).

Raman microspectroscopy: Raman maps were collected from the whole cell area for 15 cells per condition with 1µm step size, integration time of 1 s for 1 accumulation, and a spectral resolution of ca. 1.5 cm^{-1}. The spectrometer calibration was performed with an internal silicon plate.

FT-IR microspectroscopy: Maps were collected from 15 cells for each condition (the spectral range from 900 cm^{-1} to 3800 cm^{-1}, spectral resolution of 4 cm–1 and 256 scans per spectrum)

AFM-IR nanospectroscopy: Contact resonances were selected using a 180 kHz search location and a 50 kHz half-width Gaussian filter. For spectra collection, an optical parametric oscillator (OPO) laser was used as an infrared excitation source in the range of 3500-2800 cm^{-1} and 1800-900 cm^{-1} with the 4 cm^{-1} spectral resolution. Spectra were collected from 7 points that were placed diagonally across each cell and condition by co-averaging 256 pulses of excitation. For the AFM topography and IR images collection cantilever scan rate was set to 0.05 Hz. The spatial resolution was 40nm since the measured area was 20x20µm with 500x500 measurement points. of the 20x20 µm images was 500 x 500 pixels.

IR maps corresponding to topography images were collected for selected wavenumbers with a multichip tunable quantum cascade laser as an infrared source (QCL; MIRcat-QT Daylight Solutions).

Data analysis: Raman images were analyzed using WiRE (ver. 5.3, Renishaw, United Kingdom) software. Preprocessing included cosmic ray removal, noise filtering, baseline correction, and min-max normalization on all spectra. Then the hierarchical cluster analysis (HCA) was performed to differentiate the whole cell area based on spectral profile. Euclidean distance and Ward's algorithm were used to calculate spectral distances and the individual clusters. For FT-IR data preprocessing Matlab 2017 and OPUS (ver. 7.5, Bruker Optics, Germany) software were used. Mean averaged spectra from every single cell were extracted and min-max normalization in the amide I region was performed to avoid differentiation to the sample thickness. Then the second derivative IR spectra were calculated with 13 smoothing points according to the Savitzky–Golay protocol. [6,7,8]

The areas under the selected Raman and FT-IR bands (integral intensities) were calculated using OPUS (ver. 7.5, Bruker Optics, Germany). Based on the values of integral intensities box plots were constructed to obtain information about biochemical changes within cells. The variance was performed using the statistical model (ANOVA) in the OriginPro software. Tukey's test was employed to compute significance values p.

The AFM-IR spectra were analyzed using Analysis Studio (ver. 3.14) software. Spectra were smoothed according to the Savitzky-Golay protocol with 3rd-order polynomial and 5 data points, normalized (min-max normalization) and presented as second derivative IR spectra.[9,10,11] The 3D AFM and AFM-IR images were prepared with Mountainsmap software (ver. 7.3, Digital Surf, France)

The integration ranges and ratios for box plots presented in the manuscript:

- Figure 1: Integration: total lipids [2850 cm^{-1}/1000 cm^{-1}], lipids unsaturation [1651 cm^{-1}/1440 cm^{-1}], DNA [783 cm^{-1}/1000 cm^{-1}].
- Figure 2: Integration: CB_3/CH_2[2960 cm^{-1}/2854 cm^{-1}], cholesteryl esters [1730 cm^{-1}], total proteins [1650 cm^{-1}+1550 cm^{-1}], β-sheet/α-helix [1683 cm^{-1}/1650 cm^{-1}], phospholipids [1240 cm^{-1}], carbohydrates [1055 cm^{-1}+1022 cm^{-1}].

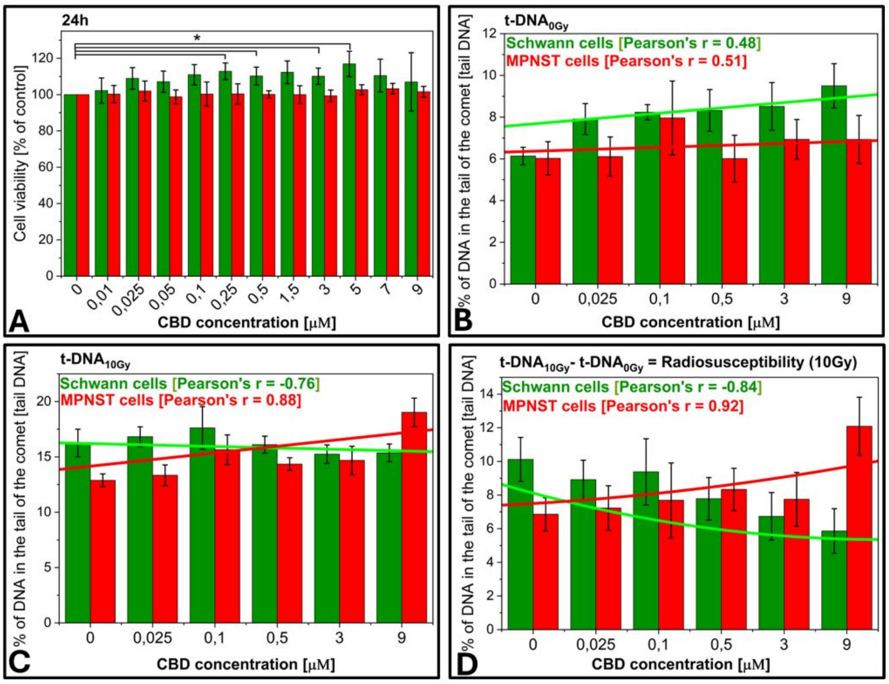

Figure S4. (A) Results of MTS test for twelve CBD concentrations (*p<0.05). (B) Results of comet assay for selected CBD concentrations. (C) Results of comet assay for selected CBD concentrations and 10Gy irradiation dose. (D) Calculated radiosusceptibility for 10Gy irradiation dose.

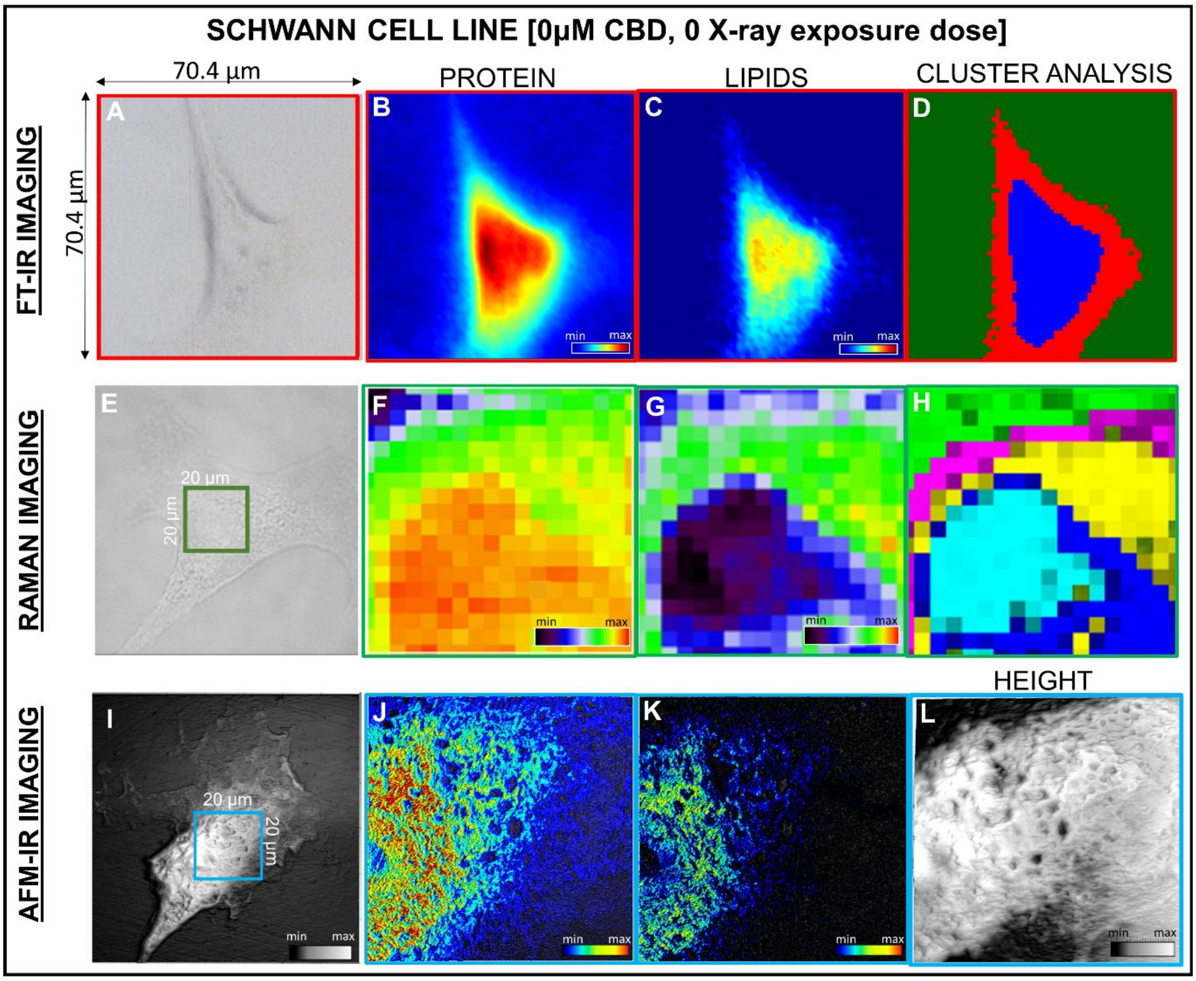

Figure S5. Comparison of spatial resolution achieved using FT-IR, Raman and AFM-IR imaging techniques. FT-IR imaging: sampling density 1.1µm x 1.1µm, (A) Microphotograph (36x objective, transmission mode, NA=0.5) with marked measurement area; (B) distribution of proteins (1650 cm^{-1}) and (C) lipids (cholesteryl esters, 1730 cm^{-1}) Cluster analysis (D) differentiates cell nuclei (blue), cytoplasm (red) and background (green). Raman imaging: sampling density 1 µm x 1µm, (E) Microphotograph (60x, immersive objective, NA=1.0) with marked measurement area; (F) distribution of proteins (1000 cm^{-1}) and (G) lipids (2850 cm^{-1}). Cluster analysis (H) differentiates cell nuclei (light blue), nucleus (dark blue), endoplasmatic reticulum (yellow), cytoplasm (pink) and cell membrane (green).

AFM-IR imaging: sampling density 40nm, (I) AFM cell topography with marked measurement area; (J) distribution of proteins (1650 cm^{-1}) and (K) lipids (cholesteryl esters, 1730 cm^{-1}). (L) detailed topography of the imaged area by AFM-IR technique.

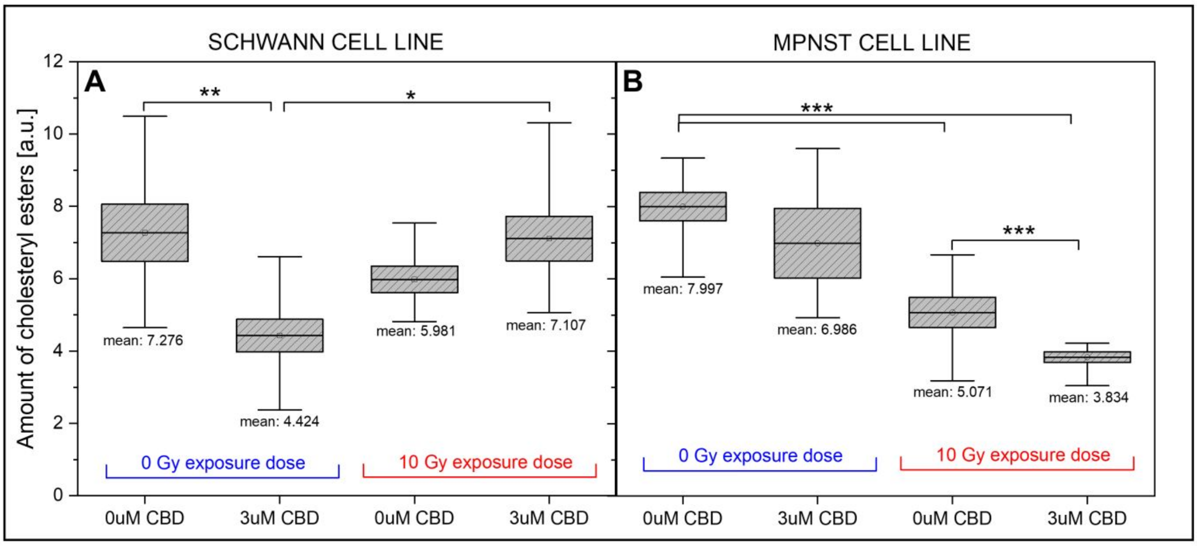

Figure S6. Calculations of integral intensities for 1730cm^{-1} band related to cholesteryl esters based on AFM-IR spectra. The experimental conditions were selected based on chemical maps presented on Figure 4 and Figure 5 to prove the differences in the local distribution of this molecules. Box diagrams are presented with mean values, standard deviation and min-max range. The variance was performed using the statistical model (ANOVA) with significance values p*<0.05, p**<0.01 and p***<0.001.

Table S1. Raman band positions observed in Raman spectra of the Schwann and MPNST cell lines with their assignment to biomolecules.[12-16]

POSITION [CM^{-1}]	ASSIGNMENT TO BIOMOLECULES AND VIBRATIONAL MODES
**3060**	Unsaturated fatty acids; ν(=C-H)
**2936**	Lipids, proteins; ν(C-H)
**2874**	Lipids, proteins; ν(C-H)-CH_2
**2850**	Long chain fatty acids; $\nu_s(CH_2)$
**1651**	Proteins (amide I); ν(C=O) and δ(N-H)
**1575**	Tyrosine
**1440**	Proteins, lipids; δ(CH2), δ(CH_3)
**1340**	A (nucleic acids); δ(CH)<br>Proteins; δ(CH)<br>Carbohydrates; δ(CH)
**1300**	Lipids; τCH_2-CH_3
**1204**	Tyrosine, Phenyloalanine, Tryptophan, Hydroxyproline (proteins); τ(CH_2)
**1125**	Lipids (trans in acyl backbone); ν(C-C)<br>Cyt; ν(C-N)<br>Proteins; ν(C-O)<br>Carbohydrates; ν(C-O)
**1081**	Phospholipids; ν(PO_2^-)<br>Lipids (trans in acyl backbone); ν(C-C)
**1000**	Phenylalanine (symmetric ring breathing)
**783**	DNA; ν_{as}(O-P-O)<br>Uracyl, Thymine, Cytosine (nucleic acids)
**640**	Tyrosine
**620**	Phenylalanine

ν – stretching mode, as – asymmetric, s – symmetric; δ – in-plane deformations; τ – twisting;

Table S2. IR band positions observed in second derivatives infrared spectra of the Schwann and MPNST cell lines with their assignment to biomolecules. [17-22]

POSITION [CM^{-1}]	ASSIGNMENT TO BIOMOLECULES AND VIBRATIONAL MODES
**3012,3020**	Unsaturated fatty acids; ν(=C-H)
**2960**	Proteins, lipids; $\nu_{as}(CH_3)$
**2923,2920**	Lipids and proteins; $\nu_{as}(CH_2)$
**2877**	Proteins, lipids, nucleic acids; $\nu_s(CH_3)$
**2880**	Terminal CH3 group in acyl chains (lipids); ν(CH)
**2850/2866**	Long chain fatty acids; $\nu_s(CH_2)$
**1740**	Triacylglycerols; $\nu_{ester}(C{=}O)$
**1728**	Cholesterol esters; $\nu_{ester}(C{=}O)$
**1720**	Fatty acids; ν(C=O)<br>Base pair (B-DNA); ν(C=O)
**1683**	β-turns in proteins (amide I); ν(C=O) and δ(N-H)<br>Guanine (DNA); ν(C=O) and ν(C=C)
**1660-1650**	α-Helices in proteins (amide I); ν(C=O) and δ(N-H)
**1625**	β-sheet in proteins (amide I); ν(C=O) and δ(N-H)
**1540-1545**	Proteins (amide II); δ(N-H) and ν(C-N)
**1514**	Tyrosine (proteins); ν(CC) of the Tyrosine ring<br>Cytosine (methylated DNA); in-plane vibrations of the ring
**1463**	Proteins; $\delta(CH_2, CH_3)$<br>Cytosine (DNA); δ(NH), ν(CC)
**1444**	Lipids; $\delta(CH_2, CH_3)$
**1386**	Free fatty acids; $\nu_s(COO^-)$<br>Free amino acids; $\nu_s(COO^-)$
**1260-1220**	Nucleic acids, phospholipids, phosphoproteins; $\nu_{as}(PO_2^-)$
**1170-1164**	Fatty acids and cholesterol esters; ν(C-O)
**1150-1153**	Glycogen; ν_{as}(CO-O-C)<br>Polysaccharides; ν_{as}(CO-O-C)
**1120-1104**	Ribose (RNA); ν(C-O)<br>Polysaccharides; ν(CC-OC)
**1090-1084**	Nucleic acids; $\nu_s(PO_2^-)$<br>Phospholipids; $\nu_s(PO_2^-)$<br>Glycogen; ν(C-C)
**1065-1044**	Carbohydrates, glycogen; ν(C–O)
**960**	DNA; ν(C-C)

ν – stretching mode, as – asymmetric, s – symmetric; δ – in-plane deformations;

Table S3. The correlation of integral intensities mean values for Raman spectra calculated for individual cells (N=15). The graphical representation can be found in Figure 1 on the manuscript.

		CBD [μM]	TOTAL LIPIDS	UNSATURATION	DNA
Schwann cell line	CBD	0	45.038	4.907	0.00435
		0.025	52.646	5.411	0.00588
		0.1	55.260	5.912	0.00615
		0.5	55.320	5.545	0.00568
		3	57.430	5.140	0.00449
		9	61.805	5.414	0.00423
	CBD+10Gy	0	67.655	5.644	0.00273
		0.025	65.530	5.028	0.00520
		0.1	67.422	4.392	0.00366
		0.5	65.885	3.828	0.00321
		3	65.986	3.839	0.0046
		9	57.067	3.965	0.00452
MPNST cell line	CBD	0	54.309	7.069	0.00597
		0.025	57.854	7.017	0.00411
		0.1	48.682	6.819	0.00715
		0.5	57.449	6.476	0.00507
		3	47.418	6.341	0.00592
		9	47.244	7.365	0.00507
	CBD+10Gy	0	53.199	7.828	0.00558
		0.025	54.355	5.619	0.00628
		0.1	54.279	6.763	0.00499
		0.5	60.123	7.102	0.00429
		3	57.265	5.449	0.00435
		9	61.013	5.214	0.00400

Table S4. The correlation of integral intensities mean values for FT-IR spectra calculated for individual cells (N=15). The graphical representation can be found in Figure 2 on the manuscript.

		CBD [μM]	CH_3/ CH_2	CHOLESTERYL ESTERS	TOTAL PROTEINS	β-SHEET/ α-HELIX	PHOSPHO-LIPIDS	CARBOHYDRATES
Schwann cell line	CBD	0	1.085	2.421	16.423	0.0902	1.327	0.371
		0.025	0.920	2.087	14.443	0.1068	1.429	0.344
		0.1	0.985	2.269	14.404	0.0859	1.375	0.396
		0.5	1.055	2.202	13.787	0.0834	1.385	0.453
		3	0.847	1.876	13.321	0.0729	1.879	0.458
		9	0.925	2.306	13.928	0.0921	1.622	0.884
	CBD+10Gy	0	0.881	2.844	16.423	0.0877	1.402	0.005734
		0.025	0.887	2.218	13.729	0.0976	1.179	0.008105
		0.1	0.833	1.963	15.222	0.0894	1.585	0.008931
		0.5	1.012	2.758	14.861	0.0901	1.501	0.007743
		3	0.926	2.828	14.755	0.0488	1.335	0.040900
		9	0.835	2.762	15.268	0.0915	1.346	0.058900
MPNST cell line	CBD	0	0.879	1.943	16.213	0.0845	1.709	0.579
		0.025	0.977	1.468	15.383	0.0700	1.797	0.548
		0.1	1.0724	2.254	15.892	0.0622	1.711	0.425
		0.5	0.872	1.640	16.541	0.0753	1.641	0.490
		3	0.651	2.231	13.782	0.0716	1.181	0.591
		9	0.824	2.175	16.671	0.0736	1.186	0.413
	CBD+10Gy	0	1.389	2.079	15.499	0.0722	1.361	0.715
		0.025	0.833	1.989	16.165	0.0639	1.660	0.677
		0.1	0.932	2.029	15.992	0.0750	1.673	0.313
		0.5	0.863	1.885	16.589	0.0679	1.541	0.305
		3	1.068	2.521	15.832	0.0733	1.191	0.358
		9	0.850	2.465	16.342	0.0858	1.363	0.399

References:

(1) Ramos-Guerreo, L; Montalvo, G; Cosmi, M; Garcia-Ruiz, C; Ortega-Ojeda, E. F Classification of Various Marijuana Varieties by Raman Microscopy and Chemometrics. Toxics. 2022, 10(3).

(2) Tay, L.L.; Hulse, J.; Paroli, M.R. FTIR and Raman Spectroscopic Characterization of Cannabinoids Can. Journal of Chem. 2022, 10(100)

(3) Sanchez, L.; Baltensperger, D.; Kurouski, D. Raman-Based Differentiation of Hemp, Cannabidiol-Rich Hemp, and Cannabis. Anal Chem. 2020, 92(11): 7733-7737

(4) Geskovski, N.; Stefkov, G.; Gigopuli, O.; Stefov, S.; Huck, W. C.; Makreski, P. Mid-infrared spectroscopy as process analytical technology tool for estimation of THC and CBD content in Cannabis flowers and extracts. SAA: Mol. Biomol. Spectr. 2021, 251, 119422

(5) Geskovski, N.; Stefkov, G.; Gigopuli, O.; Stefov, S.; Huck, W. C.; Makreski, P. Mid-infrared spectroscopy as process analytical technology tool for estimation of THC and CBD content in Cannabis flowers and extracts. SAA: Mol. Biomol. Spectr. 2021, 251, 119422

(6) Chrabaszcz, K.; Meyer, T.; Bae, H.; Schmitt, M.; Jasztal, A.; Smeda, M.; Stojak, M.; Popp, J.; Malek,

K.; Marzec, K. M. Comparison of Standard and HD FT-IR with Multimodal CARS/TPEF/SHG/FLIMS Imaging in the Detection of the Early Stage of Pulmonary Metastasis of Murine Breast Cancer. **2020**.

(7) Kujdowicz, M.; Placha, W.; Mech, B.; Chrabaszcz, K.; Okoń, K.; Malek, K. In Vitro Spectroscopy-Based Profiling of Urothelial Carcinoma: A Fourier Transform Infrared and Raman Imaging Study. *Cancers 2021, Vol. 13, Page 123* **2021**, *13* (1), 123.

(8) Chrabaszcz, K.; Jasztal, A.; Smęda, M.; Zieliński, B.; Blat, A.; Diem, M.; Chlopicki, S.; Malek, K.; Marzec, K. M. Label-Free FTIR Spectroscopy Detects and Visualizes the Early Stage of Pulmonary Micrometastasis Seeded from Breast Carcinoma. *Biochim. Biophys. Acta - Mol. Basis Dis.* **2018**, *1864* (11), 3574–3584.

(9) Piergies, N.; Mathurin, J.; Dazzi, A.; Deniset-Besseau, A.; Oćwieja, M.; Paluszkiewicz, C.; Kwiatek, W. M. IR Nanospectroscopy to Decipher Drug/Metal Nanoparticle Interactions: Towards a Better Understanding of the Spectral Signal Enhancement and Its Distribution. *Appl. Surf. Sci.* **2023**, *609*, 155217.

(10) Piergies, N.; Dazzi, A.; Deniset-Besseau, A.; Mathurin, J.; Oćwieja, M.; Paluszkiewicz, C.; Kwiatek, W. M. Nanoscale Image of the Drug/Metal Mono-Layer Interaction: Tapping AFM-IR Investigations. *Nano Res.* **2020**, *13* (4), 1020–1028.

(11) Paluszkiewicz, C.; Piergies, N.; Chaniecki, P.; Rękas, M.; Miszczyk, J.; Kwiatek, W. M. Differentiation of Protein Secondary Structure in Clear and Opaque Human Lenses: AFM – IR Studies. *J. Pharm. Biomed. Anal.* **2017**, *139*, 125–132..

(12) Bik, E.; Dorosz, A.; Mateuszuk, L.; Baranska, M.; Majzner, K. Fixed versus Live Endothelial Cells: The Effect of Glutaraldehyde Fixation Manifested by Characteristic Bands on the Raman Spectra of Cells. *Spectrochim. Acta Part A Mol. Biomol. Spectrosc.* **2020**, *240*, 118460.

(13) Majzner, K.; Chlopicki, S.; Baranska, M. Lipid Droplets Formation in Human Endothelial Cells in Response to Polyunsaturated Fatty Acids and 1-Methyl-Nicotinamide (MNA); Confocal Raman Imaging and Fluorescence Microscopy Studies. *J. Biophotonics* **2016**, *9* (4), 396–405.

(14) Prescott, B.; Steinmetz, W.; Thomas, G. J. Characterization of DNA Structures by Laser Raman Spectroscopy. *Biopolymers* **1984**, *23* (2), 235–256.

(15) Harvey, T. J.; Hughes, C.; Ward, A. D.; Faria, E. C.; Henderson, A.; Clarke, N. W.; Brown, M. D.; Snook, R. D.; Gardner, P. Classification of Fixed Urological Cells Using Raman Tweezers. *J. Biophotonics* **2009**, *2* (1–2), 47–69.

(16) Jen, C. P.; Huang, C. Te; Chen, Y. S.; Kuo, C. T.; Wang, H. C. Diagnosis of Human Bladder Cancer Cells at Different Stages Using Multispectral Imaging Microscopy. *IEEE J. Sel. Top. Quantum Electron.* **2014**, *20* (3).

(17) Miller, L. M.; Bourassa, M. W.; Smith, R. J. FTIR Spectroscopic Imaging of Protein Aggregation in Living Cells. *Biochim. Biophys. Acta* **2013**, *1828* (10), 2339.

(18) Sahu, R. K.; Argov, S.; Salman, A.; Huleihel, M.; Grossman, N.; Hammody, Z.; Kapelushnik, J.; Mordechai, S. Characteristic Absorbance of Nucleic Acids in the Mid-IR Region as Possible Common Biomarkers for Diagnosis of Malignancy. *Technol. Cancer Res. Treat.* **2004**, *3* (6), 629–638.

(19) Staniszewska, E.; Malek, K.; Baranska, M. Rapid Approach to Analyze Biochemical Variation in Rat Organs by ATR FTIR Spectroscopy. *Spectrochim. Acta - Part A Mol. Biomol. Spectrosc.* **2014**, *118*, 981–986.

(20) Wiercigroch, E.; Staniszewska-Slezak, E.; Szkaradek, K.; Wojcik, T.; Ozaki, Y.; Baranska, M.; Malek, K. FT-IR Spectroscopic Imaging of Endothelial Cells Response to Tumor Necrosis Factor-α: To Follow Markers of Inflammation Using Standard and High-Magnification Resolution. *Anal. Chem.* **2018**, *90* (6), 3727–3736.

(21) Banyay, M.; Sarkar, M.; Gräslund, A. A Library of IR Bands of Nucleic Acids in Solution. *Biophys. Chem.* **2003**, *104* (2), 477–488.

(22) Whelan, D. R.; Bambery, K. R.; Heraud, P.; Tobin, M. J.; Diem, M.; McNaughton, D.; Wood, B. R. Monitoring the Reversible B to A-like Transition of DNA in Eukaryotic Cells Using Fourier Transform Infrared Spectroscopy. *Nucleic Acids Res.* **2011**, *39* (13), 5439–5448.
